# Structure–property relationships in a graphene oxide-reinforced chitosan–carboxymethylcellulose–alginate composite for wound dressing applications

**DOI:** 10.1039/d6ra09378g

**Published:** 2026-10-29

**Authors:** Przemysław Sareło, Maria Wiśniewska-Wrona, Monika Sikora, Magdalena Pajączkowska, Joanna Nowicka, Yuriy Gerasymchuk, Maria Luisa Saladino, Patrizia Cancemi, Martina Maria Calvino, Giuseppe Lazzara, Maria Szymonowicz, Beata Sobieszczańska, Magdalena Wawrzyńska

**Affiliations:** a Pre-clinical Research Center, Wrocław Medical University Karola Marcinkowskiego 1 50-368 Wrocław Poland p.sarelo@umw.edu.pl; b Department of Biomedical Engineering, Faculty of Fundamental Problems of Technology, Wrocław University of Science and Technology Wybrzeże S. Wyspiańskiego 27 50-370 Wrocław Poland; c Centre for the Circular Economy, ŁUKASIEWICZ Research Network – Łódź Institute of Technology Marii Skłodowskiej-Curie 19/27 90-570 Łódź Poland; d Department of Microbiology, Faculty of Medicine, Wrocław Medical University Tytusa Chałubińskiego 4 50-368 Wrocław Poland; e Division of Optical Spectroscopy, Institute of Low Temperature and Structure Research, Polish Academy of Sciences Okólna 2 50-422 Wrocław Poland; f Department of Biological, Chemical and Pharmaceutical Sciences and Technologies (STeBiCeF), University of Palermo Viale Delle Scienze, Bld. 16 and 17 90128 Palermo Italy; g Consiglio Nazionale Delle Ricerche, Istituto per i Processi Chimico Fisici Viale Ferdinando Stagno d'Alcontres 37 98158 Messina Italy; h Department of Physics and Chemistry “Emilio Segrè”, University of Palermo Viale Delle Scienze Bld. 17 90128 Palermo Italy

## Abstract

Multifunctional wound dressings require polysaccharide-based biomaterials combining structural integrity, antimicrobial activity, and cytocompatibility. In this study, graphene oxide (GO)-functionalized chitosan–carboxymethylcellulose–alginate (ChitCMCAlgGO) sponge scaffolds were developed to investigate the role of GO in modulating the molecular organization and functional properties of a multicomponent polysaccharide network. The composites were characterized using SEM, FT-IR spectroscopy, solid-state ^13^C CP/MAS NMR with T_1_ρH and T_1_ρC relaxation analyses, mechanical testing, fluid-handling evaluation, antimicrobial and antibiofilm assays, and cytocompatibility assessment according to ISO 10993. GO incorporation enhanced the mechanical performance of the scaffolds, increasing tensile strength from 0.032 to 0.122 MPa and maximum tensile force from 0.93 to 3.48 N, while improving elongation at maximum stress from 37.6% to 110%. Spectroscopic and NMR analyses revealed that GO did not modify the chemical structure of the polysaccharide components but likely promoted non-covalent interactions and altered polymer-chain dynamics, resulting in increased scaffold density and reduced porosity. Despite a moderate decrease in fluid uptake, GO-functionalized scaffolds maintained high absorption capacity (34.2 g g^−1^ after 24 h). Moreover, GO improved antimicrobial activity against wound-associated pathogens and preserved cytocompatibility. These findings demonstrate that GO functions as a structural nanofiller regulating polysaccharide network organization, providing a rational strategy for designing advanced carbohydrate-based wound dressing materials.

## Abstract 2

Multifunctional wound dressings require polysaccharide-based biomaterials combining structural integrity, antimicrobial activity, and cytocompatibility.

## Graphical abstract

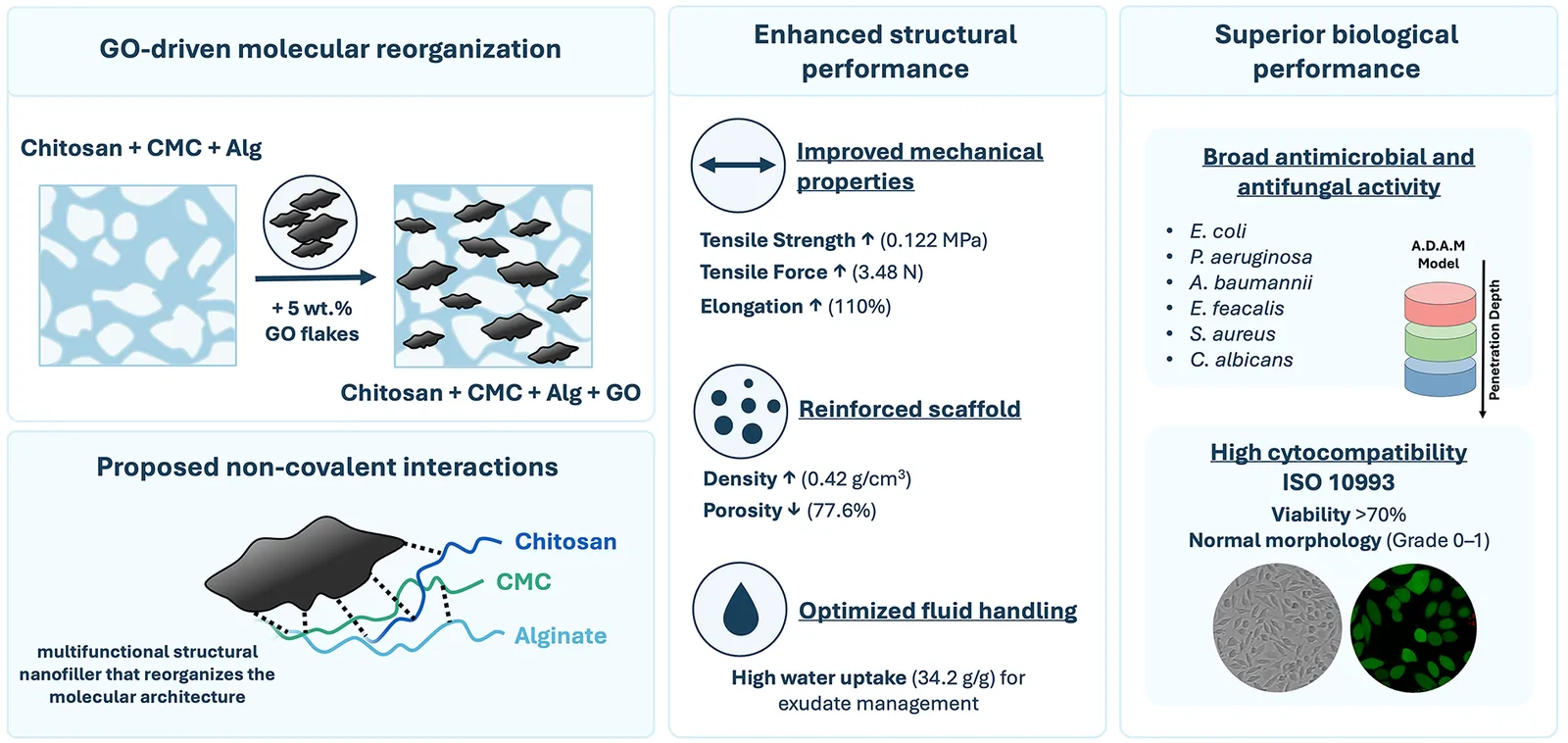

## 1 Introduction

Chronic wounds represent a major and growing healthcare challenge, affecting millions of patients worldwide and imposing a substantial socioeconomic burden on healthcare systems.^1–4^ Their impaired healing is frequently associated with diabetes, vascular disorders, aging, and persistent inflammation,^5–10^ while bacterial colonization and biofilm formation further delay tissue regeneration and markedly reduce the efficacy of conventional antimicrobial therapies.^11–15^ Consequently, there is an urgent need for multifunctional wound dressings capable of simultaneously providing a favorable environment for tissue repair while effectively preventing or controlling microbial infections.

Natural polysaccharides have emerged as attractive candidates for advanced wound dressings owing to their excellent biocompatibility, biodegradability, and structural resemblance to the extracellular matrix.^16–19^ Among them, chitosan, sodium alginate, and carboxymethylcellulose have received particular attention. Chitosan exhibits intrinsic antimicrobial and hemostatic properties, alginate possesses outstanding fluid absorption capacity and forms hydrogels that maintain a moist wound environment, whereas carboxymethylcellulose improves mechanical integrity and water retention.^20–25^ The combination of these three polymers enables the fabrication of multifunctional composite matrices with complementary biological and physicochemical properties.^26,27^

Despite these advantages, polysaccharide-based dressings remain limited by their relatively poor mechanical performance and insufficient antimicrobial activity, particularly against multidrug-resistant and biofilm-forming pathogens. Nanostructured carbon materials have therefore attracted increasing interest as reinforcing components capable of overcoming these limitations. Among them, graphene oxide (GO) is particularly promising due to its exceptionally high specific surface area, abundance of oxygen-containing functional groups, and ability to establish multiple non-covalent interactions with surrounding polymers.^28–31^ In addition to its reinforcing effect, GO has been shown to exert antimicrobial activity through membrane disruption, oxidative stress generation, and physical interactions with microbial cells.^19,32^

Beyond its antimicrobial properties, GO can profoundly influence the molecular organization of polymeric matrices. Through hydrogen bonding and electrostatic interactions, GO has the potential to modulate polymer-chain mobility, network architecture, porosity, and mechanical behavior.^33–35^ These structural changes are expected to affect not only the physicochemical characteristics of the material but also its biological performance, including antimicrobial efficacy, fluid management, and cellular response. Understanding these structure–property relationships is therefore essential for the rational design of advanced wound dressings.

Previous studies, including recently reported GO-based chitosan and carboxymethylcellulose dressings, have demonstrated the potential of GO to improve the antimicrobial activity and physicochemical properties of wound dressings.^19,32,36^ However, further investigation is needed to better understand the relationships between GO incorporation, the structural organization and molecular dynamics of multicomponent polysaccharide networks, and their resulting functional properties. In particular, studies combining physicochemical and spectroscopic characterization with antimicrobial, antibiofilm, and cytocompatibility assessments remain limited. Building upon our earlier findings, the present study investigates these relationships in GO-reinforced chitosan–carboxymethylcellulose–alginate sponge dressings by integrating morphological, mechanical, spectroscopic, microbiological, and *in vitro* biological analyses. Thus, we developed GO-functionalized chitosan–carboxymethylcellulose–alginate sponge dressings intended for the management of infected chronic wounds. The materials were comprehensively characterized by combining physicochemical, structural, microbiological, and biological analyses. Mechanical performance, fluid-handling properties, pore architecture, FT-IR spectroscopy, and solid-state NMR relaxation measurements were employed to investigate the influence of GO on the organization and molecular dynamics of the polymer network. Antimicrobial and antibiofilm activities were evaluated against clinically relevant microorganisms, whereas cytocompatibility was assessed according to ISO 10993 using indirect and direct *in vitro* assays.

It was hypothesized that GO acts not merely as an antimicrobial additive but as a multifunctional structural nanofiller capable of reorganizing the molecular architecture of the ternary polysaccharide network. Such structural reorganization was expected to simultaneously enhance mechanical performance, modulate fluid transport, improve antimicrobial efficacy, and preserve cytocompatibility. By integrating molecular-level characterization with comprehensive biological evaluation, the present study provides new insight into the structure–property–function relationships governing GO-modified polysaccharide wound dressings and supports their development as next-generation biomaterials for chronic wound management.

## 2 Materials and methods

### 2.1. Preparation of GO

GO was prepared using a modified Brodie's oxidation protocol, following the procedure previously reported in our earlier study^19,32^ and based on the approach described by Szabó *et al.*^37^ Synthetic graphite powder (Sigma-Aldrich, St. Louis, MO, USA) was subjected to repeated oxidative treatment in fuming nitric acid (HNO_3_, Merck, Darmstadt, Germany) with potassium chlorate (KClO_3_, Sigma-Aldrich) as the oxidizing agent. The oxidation process was performed in four consecutive cycles at 60–80 °C under continuous mechanical stirring, with each cycle lasting 24–48 h.

After completion, the suspension was rinsed with deionized water (Milli-Q, Merck Millipore, Burlington, MA, USA) and washed with 10% hydrochloric acid (HCl, Merck) to remove residual inorganic impurities. The material was subsequently washed with deionized water until neutral pH was achieved. The purified product was dried at 40 °C in a forced-air oven (Binder ED 53, Tuttlingen, Germany) for 72 h.

For dispersion preparation, dried GO powder was suspended in deionized water and exfoliated using a high-power ultrasonic homogenizer (UZDN-M-900-T, Akademprylad, Lviv, Ukraine; 20 kHz, 900 W) operated at 70% amplitude for 2 h. To remove larger agglomerates, the suspensions were centrifuged twice at 8000 rpm using a refrigerated centrifuge (5804R, Eppendorf, Hamburg, Germany) for 10 min prior to further use.

### 2.2. Fabrication of biopolymers matrices and incorporation of GO

#### 2.2.1. Materials and reagents

The following materials were used for the fabrication of the polymer matrices: high-quality chitosan HQG 110 TM 5962 (Chit, Primex, Siglufjörður, Iceland), sodium carboxymethylcellulose (CMC, Sigma-Aldrich, cat. no. 419303), and sodium alginate (Alg, Sigma-Aldrich, cat. no. A2033) derived from brown algae. Supporting reagents included lactic acid (88%, analytical grade, Chempur, Piekary Śląskie, Poland) and glycerin meeting Ph. Eur. requirements (Riedel-de Haën, Seelze, Germany). The detailed physicochemical parameters of the starting polymers, including molecular weights and purity profiles, are summarized in Table 1.

**Table 1:** Physicochemical characteristics of the starting polymers^a^

Polymer	*M*_v_/*M*_w_ (kDa)	Specific parameter	Heavy metal content
Chit	337.0	SD = 77.0%	As < 0.2; Cd < 0.2; Pb < 1.0; Zn = 0.22; Hg < 0.01
		WRV = 190.0%	
CMC	250.0	DS = 0.9	As < 0.4; Cd < 0.014; Pb < 0.102; Hg < 0.02; Cr = 0.44
Alg	495.0^b^	*η* = 56.0 cP pH = 7.0 M/G = 1.9^b^	As < 0.4; Cd < 0.01; Pb < 0.1; Hg < 0.02; Cr = 0.59

a Where: *M*_v_ – viscosity-average molecular weight; *M*_w_ – weight-average molecular weight; SD – degree of deacetylation; DS – degree of substitution; *η* – dynamic viscosity; WRV – water retention value.

b Literature-reported values for the same commercial alginate.

#### 2.2.2. Physicochemical analysis of constituent materials

The properties of the starting polymers were verified according to standardized internal procedures of ŁUKASIEWICZ Research Network – Łódź Institute of Technology (Łódź, Poland), which are detailed further below.

Heavy metal analysis was determined using high-resolution atomic absorption spectrometry (Contra 700, Analytik Jena, Jena, Germany) *via* electrothermal atomic absorption spectrometry (ET AAS), flame atomic absorption spectrometry (FAA), and cold vapor atomic absorption spectrometry (CVAAS), according to internal procedure NL-13, edition V. Chit was characterized by determining the average molecular weight (*M*_v_) *via* the viscometric method using a dilution viscometer (capillary no. 1, *K* ≈ 0.01), according to the internal standard procedure SPR/BPB/5, while the degree of deacetylation (SD) was measured using the first-derivative UV spectrophotometry method (UNICAM 5425, Thermo Fisher Scientific, Waltham, MA, USA) in accordance with procedure SPR/BLF/21. For CMC, the weight-average molecular weight (*M*_w_) was verified as specified by the manufacturer, and its degree of substitution (DS) was determined *via* the acidimetric titration method. The dynamic viscosity (*η*) of the Alg solution was measured at 25 ± 1.0 °C using a Brookfield DV-II digital viscometer equipped with Rheocalc V3.1-1 software (Brookfield Engineering Laboratories, Middleboro, MA, USA); the measurements were performed using a CPE-41 cone with a 2.0 mL sample volume and a 15 min thermostating period, according to internal procedure SPR/BPB/25. The molecular-weight characteristics of Alg (*M*_w_ = 495 kDa; *M*_n_ = 249 kDa; *M*_w_/*M*_n_ = 2.0) and its M/G ratio (1.9) were adopted from previously published characterization of the same commercial product (*i.e.*, Sigma-Aldrich, cat. no. A2033). In the cited study, *M*_w_ and *M*_n_ were determined by GPC using an Ultrahydrogel guard column followed by Ultrahydrogel 2000, Ultrahydrogel 250, and Ultrahydrogel 120 columns, with 0.1 M NaNO_3_ as the mobile phase at 0.6 mL min^−1^ and 35 °C, while the M/G ratio was determined by ^1^H NMR analysis of the anomeric region.^38^

The swelling capacity, expressed as water retention value (WRV), of raw Chit was determined gravimetrically according to internal procedure SPR/BPB/14 after 20 h of immersion in water and subsequent centrifugation at 4000 rpm for 10 min.

#### 2.2.3. Preparation of composite materials with GO incorporation

Porous sponge materials were fabricated from a ternary polymer blend composed of Chit, CMC, and Alg. Chit was dissolved at a concentration of 1.0 wt% in a 0.45 wt% aqueous lactic acid solution to obtain chitosan lactate, whereas CMC and Alg were each dissolved at a concentration of 1.0 wt% in microbiologically pure water. The polymer solutions were subsequently combined at a dry weight ratio of 40 : 20 : 20 (Chit : CMC : Alg) and homogenized using a T50 basic homogenizer (IKA-Werke, Staufen, Germany) equipped with a G45G dispersing tool (particle size range 10–100 µm) at 4000–6000 rpm for 1–2 h at 20–25 °C to obtain a homogeneous polymer blend.

For the preparation of GO-functionalized dressings (ChitCMCAlgGO), GO was added at 5 wt% relative to the total dry polymer content, followed by an additional homogenization step at 3000 rpm to ensure uniform dispersion of GO throughout the polymer matrix. Glycerol was subsequently incorporated as a plasticizer at a polymer-to-glycerol dry weight ratio of 1 : 0.5. The GO concentration was selected based on previous reports describing stable GO dispersion and favorable mechanical and biological performance in polysaccharide-based composites.^19,32^

The resulting polymer suspensions were cooled, cast into molds, and frozen at −42 °C for 50 min. The frozen samples were then lyophilized using an ALPHA 2–4 LSC freeze dryer (Martin Christ, Osterode am Harz, Germany). Primary drying was carried out between −22 °C and −15 °C under a vacuum of 0.10–0.045 mbar for 16 h, followed by secondary drying at 15 °C under a vacuum of 0.006 mbar for approximately 3 h. This freeze-drying protocol produced highly porous sponge dressings with a homogeneous internal architecture.

The obtained materials, designated ChitCMCAlg and ChitCMCAlgGO, were cut into 1 × 1 cm specimens for further physicochemical, microbiological, and biological investigations. Prior to biological testing, all samples were sterilized by gamma irradiation (25 kGy) at the Institute of Nuclear Chemistry and Technology (Warsaw, Poland).

### 2.3. Physicochemical characterization of the composite materials

#### 2.3.1. Evaluation the mechanical properties

The mechanical properties of the ChitCMCAlg and ChitCMCAlgGO sponge dressings were evaluated according to standardized methods. Prior to testing, all samples were conditioned for at least 24 h at a temperature of 23 ± 1 °C and relative humidity of 50 ± 2%.

The thickness of the samples was measured in accordance with ISO 1923:1999.^39^ The measurement was performed by using a mechanical thickness gauge TILMET-64 (Łódź University of Technology, Łódź, Poland) equipped with a measuring foot of 50.5 mm in diameter. The applied pressure was 0.1 kPa, and the measurement accuracy was 0.05 mm.

The number of measurement points depended on the size and shape of the sample but was not less than five. Measurement points were distributed as far apart as possible to account for thickness variations across the entire sample. At each point, three readings were taken, the median value was selected, and the final thickness was calculated as the arithmetic mean of five median values.

Tensile properties, including maximum tensile force, tensile strength, and elongation at maximum stress, were determined using a tensile testing machine model 5544 (Instron, Norwood, MA, USA) in accordance with ISO 1798:2009.^40^ The initial grip separation was set to 10 mm, and the crosshead speed was 10 mm min^−1^. The width of the tested samples was 15 mm. Each measurement was performed in five replicates.

#### 2.3.2. Evaluation of sorption properties

The sorption properties of the developed sponge dressings were evaluated using a gravimetric method. Square samples (2 × 2 cm) were immersed in 10 mL of deionized water and incubated for predetermined time intervals (*i.e.*, 0.25, 0.5, 3, 5 and 24 h).

After incubation, samples were removed, gently blotted with filter paper to eliminate excess surface liquid, and immediately weighed. The sorption capacity was expressed as the amount of absorbed liquid per gram of dry material (g g^−1^), as well as the sorption index (*W*_s_, %), calculated according to the following equation: 
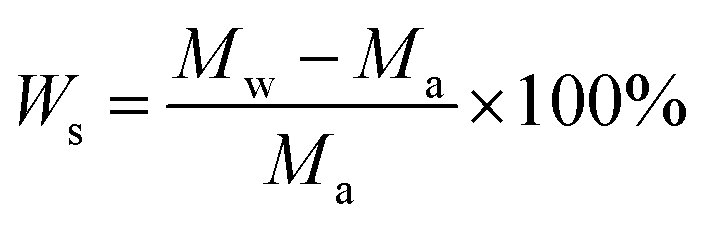
, where *M*_w_ is the mass of the swollen sample and *M*_a_ is the initial dry mass.

#### 2.3.3. Evaluation of the internal structure

The morphology and internal porous architecture of the sponge dressings were characterized using a Phenom ProX G6 Scanning Electron Microscope (SEM, Thermo Fisher Scientific, Waltham, MA, USA) equipped with a backscattered electron detector (BSD). Prior to imaging, the samples were sputter-coated with a nanometric layer of gold using a Q150RS sputter coater (Quorum Technologies Ltd, Laughton, UK). SEM observations were performed under a vacuum of 1 Pa using an accelerating voltage of 10 kV. Image acquisition and processing were carried out using Phenom ProSuite software (Thermo Fisher Scientific), enabling qualitative evaluation of the pore morphology, pore wall structure, and overall architecture of the porous dressings.

#### 2.3.4. Assessment of the density and porosity

The bulk density and total porosity of the sponge dressings were determined using an Ultrapyc 5000 Foam gas pycnometer (Anton Paar, Graz, Austria). The measurements were performed using helium gas (99.995% purity) as the displacement medium. The instrument determines the skeletal volume based on pressure changes in a calibrated chamber according to the gas expansion method. Bulk density and total porosity were subsequently calculated automatically by the instrument software from the measured skeletal volume and the geometrical dimensions of the specimens.

#### 2.3.5. FT-IR spectroscopic analysis

Fourier transform infrared (FT-IR) spectroscopy was performed to characterize the chemical structure of the individual components (Chit, CMC, Alg, and GO) as well as the developed composite dressings.

For the reference materials, Chit was dissolved in 1.0% (v/v) aqueous acetic acid, whereas CMC and Alg were dissolved separately in deionized water under magnetic stirring at room temperature until homogeneous solutions were obtained. GO was dispersed in deionized water by probe ultrasonication (20 kHz, 900 W, 70% amplitude) for 2 h, followed by centrifugation at 8000 rpm for 10 min to remove large aggregates. The resulting supernatant was used for further analyses.

FT-IR spectra of the individual components were recorded using a Nicolet 6700 FT-IR spectrometer (Thermo Fisher Scientific) equipped with a Golden Gate Mk II ATR accessory with a diamond crystal (PIKE Technologies, Fitchburg, WI, USA). Polymer solutions and the GO dispersion were deposited onto the ATR crystal and allowed to dry at room temperature for approximately 1 h prior to analysis. Spectra were collected over the 4000–400 cm^−1^ spectral range with a resolution of 4 cm^−1^, averaging 512 scans per spectrum. A background spectrum was acquired before each measurement.

The composite dressings (ChitCMCAlg and ChitCMCAlgGO) were analyzed using a Nicolet iN10 MX infrared microscope (Thermo Fisher Scientific) equipped with a MicroTip ATR accessory and a DTGS detector. Spectra were recorded over the 4000–400 cm^−1^ range at 4 cm^−1^ resolution using 512 accumulated scans from a 100 × 100 µm^2^ sampling area.

All spectra were processed using OriginPro 8.5 (OriginLab Corporation, Northampton, MA, USA). Baseline correction and Savitzky–Golay smoothing (second-order polynomial, 21-point window) were applied prior to normalization to the C–H stretching band at approximately 2930 cm^−1^.

#### 2.3.6. Solid-state NMR relaxation analysis of polymer chain dynamics

Solid-state nuclear magnetic resonance (ss-NMR) measurements were performed using a Bruker Avance II 400 spectrometer (9.4 T, Bruker BioSpin, Rheinstetten, Germany) operating at 400.15 MHz for ^1^H and 100.62 MHz for ^13^C. Samples were packed into 4 mm zirconia MAS rotors fitted with Kel-F caps.

^13^C{^1^H} CP/MAS NMR spectra were acquired at 300 K with a magic-angle spinning (MAS) frequency of 7 kHz, employing a 4.5 µs 90° proton pulse, 2 ms cross-polarization contact time, 3 s recycle delay, and 400 scans. The Hartmann–Hahn matching condition was optimized using adamantane, which also served as the external chemical shift reference (29.5 ppm).

The proton spin–lattice relaxation time in the rotating frame, T_1_ρ(H), was determined indirectly using the variable spin-lock (VSL) pulse sequence with spin-lock durations ranging from 0.1 to 7.5 ms and a 1.5 ms cross-polarization contact time. The carbon spin–lattice relaxation time in the rotating frame, T_1_ρ(C), was measured using the VSL sequence after cross-polarization with spin-lock durations ranging from 0.4 to 30 ms, while maintaining the same 1.5 ms contact time. All spectra were acquired under high-power ^1^H decoupling during signal acquisition.

### 2.4. Microbiological evaluation of antimicrobial and antibiofilm activity

#### 2.4.1. Microbial strains and culture conditions

The antimicrobial performance of the ChitCMCAlg and ChitCMCAlgGO dressings was evaluated against a representative panel of wound-associated pathogens. The following reference strains from the American Type Culture Collection (ATCC) were utilized: *Staphylococcus aureus* (ATCC 25923), *Pseudomonas aeruginosa* (ATCC 27853), *Enterococcus faecalis* (ATCC 29212), *Escherichia coli* (ATCC 25922), *Acinetobacter baumannii* (ATCC BAA1605), and the fungal pathogen *Candida albicans* (ATCC 90028).

The reference strains were stored at −80 °C in Tryptic Soy Broth (TSB, BioMaxima, Lublin, Poland) supplemented with 15% glycerol to maintain genetic stability. Prior to each experimental cycle, the strains were subcultured aerobically at 37 °C for 24–48 h on Tryptic Soy Agar (TSA, BioMaxima) for bacterial strains and Sabouraud Agar (BioMaxima) for *C. albicans*.

Standardized microbial suspensions were prepared using a densitometer DEN-1 (Grant Instruments, Argenta, Poland) to reach a turbidity of 0.5 McFarland standard. This corresponded to a concentration of approximately 1.5 × 10^8^ CFU mL^−1^ for bacteria and 1.5 × 10^6^ CFU mL^−1^ for fungi. The suspensions were prepared in TSB for bacterial cultures and Sabouraud Broth for the fungal to ensure optimal metabolic activity during the tests.

#### 2.4.2. Assessment of antimicrobial activity against planktonic cells

The impact of the composite dressings on the viability of planktonic microbial cells was evaluated using a quantitative culture-based method based on colony-forming unit (CFU) enumeration. Freshly prepared microbial suspensions, standardized to 0.5 McFarland, were diluted 10-fold in TSB for bacteria and Sabouraud Broth for the yeast strain to achieve an initial concentration of bacteria and yeast suspensions approximately 1.5 × 10^7^ CFU mL^−1^ and 1.5 × 10^5^ CFU mL^−1^, respectively.

Subsequently, the dressing samples were placed in 1 mL of the prepared microbial suspensions in sterile Eppendorf tubes and incubated aerobically at 37 °C for 24 h. Microbial cultures without the addition of any dressing served as the negative control. Following incubation, 100 µL of each suspension was collected and subjected to a series of ten-fold dilutions in sterile saline. The diluted samples were then plated onto TSA or Sabouraud Agar and incubated for another 24 h at 37 °C.

The surviving microbial population was quantified by counting the colony-forming units according to the following formula: CFU mL^−1^ = (average number of colonies) × (reciprocal of the dilution)/(plated volume), where the reciprocal of the dilution represents the inverse of the decimal dilution factor (*e.g.*, 10^4^ for a 10^−4^ dilution), and the plated volume is expressed in milliliters.

All experiments were performed in triplicate to ensure statistical reliability. The results were expressed as the mean number of CFU mL^−1^ and compared against the control group to determine the antimicrobial efficacy of the ChitCMCAlg and ChitCMCAlgGO dressings.

#### 2.4.3. Evaluation of dressing-mediated eradication of preformed biofilms

The ability of the dressings to eradicate preformed biofilms was evaluated using a static *in vitro* model on polystyrene surfaces. Briefly, microbial suspensions adjusted to 0.5 McFarland were diluted 1 : 10 in TSB (bacteria) or Sabouraud Broth (*C. albicans*) to obtain the initial inoculum. Subsequently, 1 mL of the suspension was transferred into the wells of sterile 24-well polystyrene plates (FL Medical, Equimed, Wrocław, Poland) and incubated aerobically at 37 °C for 24 h to allow for the formation of mature biofilms.

After incubation, non-adherent cells were removed by washing the wells with sterile 0.9% NaCl. Subsequently, the samples of the dressings (10 mm × 10 mm) were placed directly onto the established biofilms and moistened with 150 µL of sterile 0.9% NaCl to simulate physiological wound-like conditions. The plates were then incubated for an additional 24 h at 37 °C.

Following treatment, the dressings were carefully removed, and the remaining biofilm biomass was recovered using sterile swabs. The swabs were transferred into 1 mL of 0.5% saponin solution (Sigma-Aldrich) and vortexed for 1 min using a Microspin FV-2400 vortex mixer (Biosan, Biogenet Poland) to detach and disperse microbial cells. The resulting suspensions were serially diluted and plated onto appropriate agar media. After incubation for 24–48 h, colony-forming units (CFU mL^−1^) were determined. CFU mL^−1^ values were calculated as previously described in Section 2.4.2. Unexposed biofilms served as negative controls. All experiments were performed in triplicate.

#### 2.4.4. Antibiofilm dressing's activity measurement (A.D.A.M.)

To evaluate the capacity of the antimicrobial agents released from the composite dressings to penetrate and eradicate complex, spatial microbial structures, Antibiofilm Dressing's Activity Measurement (A.D.A.M.) model was implemented.^41^

Initially, mature biofilms were established on the surface of sterile agar discs (TSA or Sabouraud Agar, diameter 12 mm, height 5 mm) by inoculating them with 1 mL of the 10-fold diluted standardized microbial suspension and incubating for 24 h at 37 °C. To simulate the challenge of deep-seated wound infections, these biofilm-laden discs were subsequently arranged in a vertical triple-layer stack, designated as top (C – contact layer), middle (M – medium layer), and bottom (B – basal layer). The investigated ChitCMCAlg and ChitCMCAlgGO dressings were applied topically onto the uppermost disc (C) and incubated for an additional 24 h at 37 °C, while untreated stacks served as the negative control.

Following the treatment period, the spatial efficacy was quantified through metabolic viability assays using tetrazolium salts – TTC (1 mg mL^−1^ in TSB, Sigma-Aldrich) for bacteria and MTT (1 mg mL^−1^ in Sabouraud Broth, Sigma-Aldrich) for the fungal. After a 4 h incubation in the dark, the resulting formazan crystals were eluted with 1 mL of acidified isopropanol (Warchem, Zakręt, Poland) under constant agitation. The absorbance was measured spectrophotometrically at 490 nm (for TTC) and 550 nm (for MTT) by microplate reader Asys UVM 340 (Biochrom, Cambridge, United Kingdom) to determine the percentage of metabolic reduction in relation to negative control.

### 2.5. Cytocompatibility assessment

#### 2.5.1. Cell lines and cell culture conditions

Mouse fibroblast L929 cells (L929; NCTC clone 929: CCL-1, ATCC, Manassas, VA, USA) were used as standard reference cell lines for *in vitro* cytotoxicity evaluation in accordance with ISO 10993-5:2009.^42^

L929 cells were cultured in Minimum Essential Medium (MEM; Gibco, Thermo Fisher Scientific, Waltham, MA, USA) supplemented with 10% fetal bovine serum (FBS; Gibco), 4 mM l-glutamine (Gibco), 1 mM sodium pyruvate (Gibco), and a 1 : 100 antibiotic–antimycotic solution (Gibco), hereafter referred to as complete medium. Cells were maintained at 37 °C in a humidified atmosphere containing 5% CO_2_.

#### 2.5.2. Indirect contact cytotoxicity assay (extract method)

Cytotoxicity of the tested materials was evaluated using an indirect contact method based on material extracts prepared according to ISO 10993-12:2012.^43^

Material extracts of the porous, low density dressing materials were prepared at a ratio of 1 cm^2^ mL^−1^ (corresponding to approximately 15 mg mL^−1^) by incubating sterilized samples in complete culture medium at 37 °C for 24 h. After incubation, the extracts were collected and applied to cells at different dilutions (100%, 50%, 25%, and 12.5%).

L929 cells were detached using TrypLE Express Enzyme (Gibco) and seeded into 96-well plate (Techno Plastic Products AG, Trasadingen, Switzerland) at a density of 1.0 × 10^4^ cells per well in 100 µL of complete medium. After 24 h of incubation, the medium was replaced with 100 µL of the respective material extracts. Cells cultured in fresh medium served as the negative control, while sodium dodecyl sulfate (SDS) solution (Invitrogen, Thermo Fisher Scientific, Waltham, MA, USA) at concentration of 0.1 mg mL^−1^ in complete medium was used as a positive cytotoxic control. High-density polyethylene (HDPE; USP Reference Standard, Sigma-Aldrich) extract served as an additional negative control, prepared analogously to the tested materials at a surface area-to-volume ratio 1 cm^2^ mL^−1^, which corresponded to approximately 160 mg mL^−1^ for HDPE.

For the evaluation of cell morphology, cells were seeded in 24-well plates (Techno Plastic Products AG) at a density of 5 × 10^4^ cells per well and treated as described above.

#### 2.5.3. Cell viability evaluation

The 3-(4,5-dimethylthiazol-2-yl)-2,5-diphenyltetrazolium bromide (MTT) assay was used to assess cell viability and proliferation.

After 24 h exposure to material extracts, 10 µL of MTT stock solution (5 mg mL^−1^ in PBS; invitrogen) was added to each well to obtain a final concentration of 0.5 mg mL^−1^. The plate was incubated for 4 h under standard culture conditions (37 °C, 5% CO_2_). After incubation, the supernatant was carefully removed, and formazan crystals were dissolved by adding DMSO (Sigma-Aldrich). The plate was shaken on an orbital shaker at 37 °C for 15 min to ensure complete dissolution.

Absorbance was measured using an Infinite M Nano+ microplate reader (Tecan Group Ltd, Männedorf, Switzerland) at 570 nm with a reference wavelength of 630 nm. Wells containing DMSO without cells were used as blanks. Results were expressed as a percentage of the negative control (cells cultured in complete medium).

#### 2.5.4. Morphological assessment of cells

Cell morphology was documented using an inverted phase-contrast microscope (MBI 100T, mikroLAB, Lublin, Poland) equipped with a Gryphax Subra camera (Jenoptik AG, Jena, Germany), and images were acquired and processed using Gryphax Software (Jenoptik AG).

Morphological changes were assessed according to ISO 10993-5:2009 cytotoxicity grading criteria, based on the degree of cell degeneration, including changes in general cell morphology, membrane integrity, vacuolization, cell lysis, and detachment. Cytotoxicity was classified on a semi-quantitative scale (grade 0–4), where grade 0 indicates no detectable reactivity and grade 4 indicates severe cytotoxic effects with nearly complete cell destruction.

#### 2.5.5. Direct contact cytotoxicity assay

Direct cytotoxic effects were evaluated according to ISO 10993-5:2009 using a direct contact method.^42^

Cells were seeded into 12-well plate (Techno Plastic Products AG) at a density of 1 × 10^5^ cells per well in 1.5 mL of complete medium. After 24 h incubation, the tested material samples were carefully placed directly onto the confluent cell monolayer.

After 24 h of contact, cell morphology was evaluated using phase-contrast inverted microscopy. Representative images were acquired from regions located directly beneath the material samples, at the sample–cell interface, and at increasing distances within the surrounding cell monolayer.

Morphological changes were assessed according to ISO 10993-5:2009 cytotoxicity grading criteria, as described above.^42^

#### 2.5.6. Live/dead fluorescence staining

Cell viability and membrane integrity were qualitatively assessed using fluorescent live/dead staining. Cells were seeded into 4-well µ-plate (Techno Plastic Products AG) at a density of 5 × 10^4^ cells per well in 700 µL of complete medium. After 24 h incubation, the tested material samples were carefully placed directly onto the confluent cell monolayer.

After treatment (direct contact), cells were stained with calcein AM (Invitrogen) and propidium iodide (PI; Sigma-Aldrich) at final working concentrations of 2 µM and 2 µg mL^−1^, respectively. Cells were incubated for 20 min at 37 °C in the dark.

Images were acquired using a TCS SPE confocal microscope (Leica Microsystems, Wetzlar, Germany) equipped with diode lasers at excitation wavelengths of 488 nm (calcein AM) and 532 nm (propidium iodide). Image acquisition was performed using LAS AF software (Leica Microsystems).

Viable cells were identified by calcein fluorescence, while dead cells were detected based on propidium iodide uptake.

### 2.6. Statistical analysis

The statistical analysis of the obtained datasets was performed using GraphPad Prism 11 (GraphPad Software, San Diego, CA, USA).

The Shapiro–Wilk test was used to assess data normality, and Levene's test was used to evaluate homogeneity of variance. The significance level was set at 0.05.

For comparisons between two groups, data with normal distribution were analyzed using an unpaired *t*-test, whereas non-normally distributed data were analyzed using the Mann–Whitney *U* test.

For comparisons involving more than two groups, one-way analysis of variance (ANOVA) followed by an appropriate *post hoc* test (Tukey's or Dunnett's multiple comparisons test) was used for normally distributed data. For non-parametric data, the Kruskal–Wallis test followed by Dunn's multiple comparisons test was applied.

## 3 Results

### 3.1. Physicochemical characterization of the composite materials

#### 3.1.1. Mechanical properties of the dressings

The mechanical properties (*i.e.*, thickness, maximum tensile force, tensile strength and elongation at maximum stress) of the developed sponge dressings were significantly influenced by the incorporation of GO, as summarized in Table 2.

**Table 2:** Mechanical properties (*i.e.*, thickness, maximum tensile force, tensile strength and elongation at maximum stress) of ChitCMCAlg sponge dressings with and without GO

Parameter	ChitCMCAlg	ChitCMCAlgGO
Thickness ± measurement uncertainty (mm)	1.42 ± 0.36	1.90 ± 0.58
Maximum tensile force ± measurement uncertainty (N)	0.93 ± 0.09	3.48 ± 0.58
Tensile strength ± measurement uncertainty (MPa)	0.032 ± 0.003	0.122 ± 0.020
Elongation at maximum stress ± measurement uncertainty (%)	37.6 ± 9.2	110.0 ± 40.0

The GO-containing composite exhibited a greater thickness (1.90 ± 0.58 mm) compared to the GO-free ChitCMCAlg matrix (1.42 ± 0.36 mm). More importantly, a substantial enhancement in tensile performance was observed upon GO incorporation. The maximum tensile force increased from 0.931 ± 0.084 N for the GO-free material to 3.48 ± 0.58 N for the GO-modified composite. A similar trend was observed for tensile strength, which increased from 0.032 ± 0.003 MPa to 0.122 ± 0.02 MPa, indicating a nearly fourfold improvement. In addition, elongation at maximum stress increased markedly from 37.6 ± 9.2% to 110 ± 40%, suggesting a significant enhancement in material flexibility.

The improvement in tensile performance following GO incorporation may reflect the combined effects of GO-polymer interactions and changes in scaffold architecture. In particular, the increased density, reduced porosity, and altered pore morphology may influence stress distribution and deformation within the composite. The present study demonstrates the overall effect of GO incorporation but does not quantify the individual contributions of interfacial interactions and architecture-related changes. Overall, the incorporation of GO resulted in a pronounced improvement in both strength and deformability of the sponge dressings.

#### 3.1.2. Sorption properties of the dressings

The developed sponge dressings exhibited high sorption capacity, confirming their suitability for applications requiring effective fluid management – Table 3.

**Table 3:** Evaluation of the sorption capacity of wound dressing sponges based on ChitCMCAlg composite with and without GO

Measurement time (h)	ChitCMCAlg		ChitCMCAlgGO	
	Sorption index (*W*_s_, %)	Sorption capacity (g g^−1^)	Sorption index (*W*_s_, %)	Sorption capacity (g g^−1^)
0.25	3454.1 ± 232.6	34.54 ± 2.33	3122.5 ± 388.9	31.22 ± 3.89
0.5	3655.2 ± 223.0	36.55 ± 2.23	3211.5 ± 359.7	32.11 ± 3.60
3	3827.1 ± 202.4	38.27 ± 2.03	3273.2 ± 347.5	32.73 ± 3.48
5	3926.1 ± 220.0	39.26 ± 2.20	3336.4 ± 325.8	33.36 ± 3.26
24	4092.0 ± 197.1	40.92 ± 1.98	3415.9 ± 300.5	34.16 ± 3.01

At 5 h, the ChitCMCAlg matrix without GO reached a sorption index of 3926.1 ± 220.0% (39.26 ± 2.20 g g^−1^), whereas the GO-containing composite exhibited a lower value of 3336.4 ± 325.8% (33.36 ± 3.26 g g^−1^). After 24 h, the sorption capacity further increased for both materials, reaching 4092.0 ± 197.1% (40.92 ± 1.98 g g^−1^) for the GO-free matrix and 3415.9 ± 300.5% (34.16 ± 3.01 g g^−1^) for the GO-containing composite.

Although the incorporation of GO resulted in a reduction in sorption capacity at all time points, the GO-modified material still maintained high its values approximately 35 g g^−1^.

#### 3.1.3. Surface morphology and the porous architecture of the dressings with or without GO

The internal architecture of the fabricated ternary sponges was evaluated using SEM at 80× and 500× magnification (Fig. 1).

**Fig. 1:**
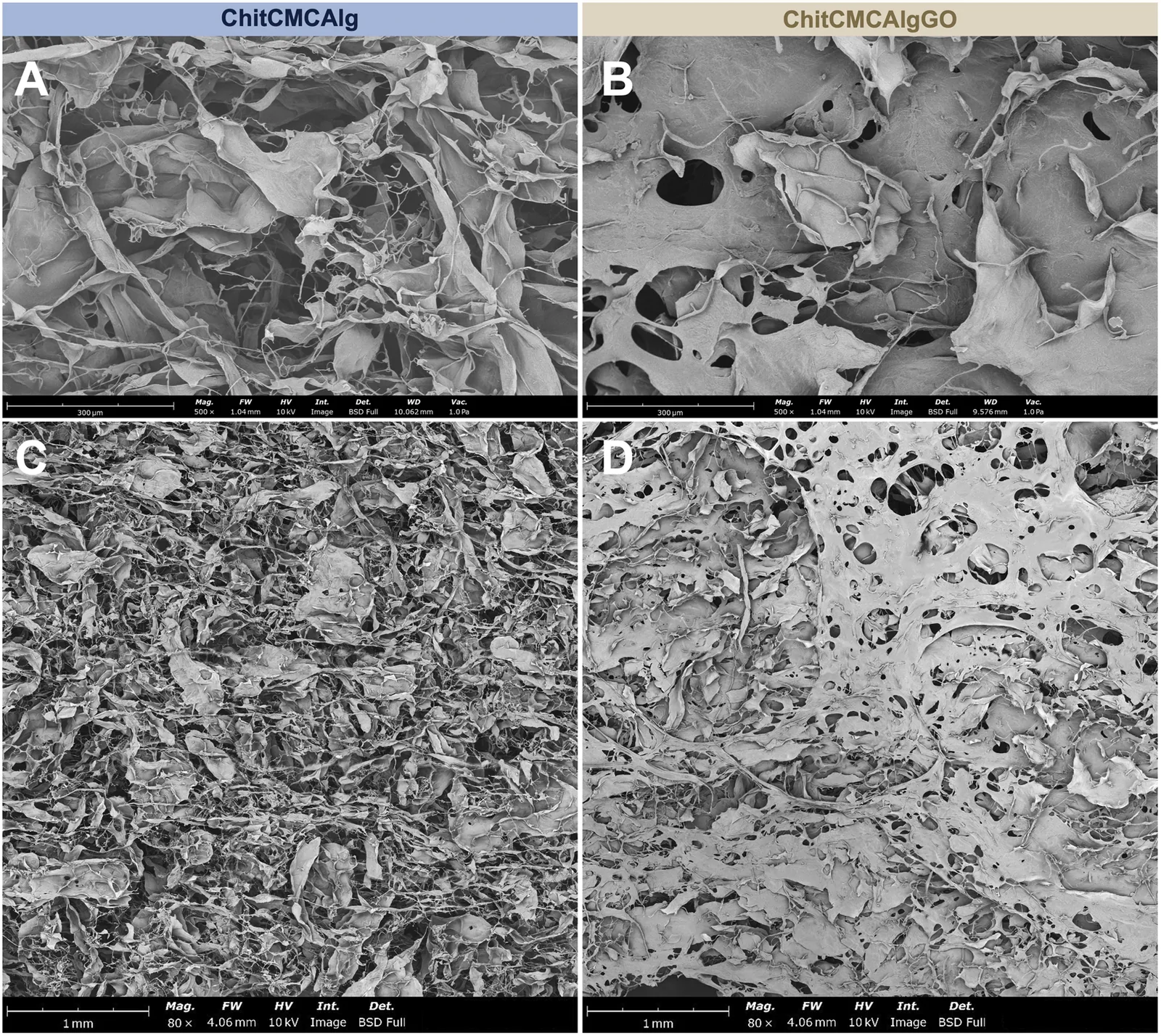
Morphological characterization of the porous sponges: (A and C) reference ChitCMCAlg matrix showing large, open voids and (B and D) ChitCMCAlgGO composite with a refined, more compact pore network. SEM images taken at (A and B) 500× and (C and D) 80× magnification. Scale bars: 300 µm and 1 mm, respectively.

The unmodified ChitCMCAlg matrix exhibited a highly porous structure characterized by large, open, and interconnected voids with relatively thin pore walls. Upon the incorporation of GO, a distinct morphological transition was observed. The functionalized sponges displayed a significantly more compact and refined internal network. The SEM images clearly show a reduction in the average pore size, and a denser arrangement of the polymer matrix compared to the raw polymer blend, indicating a high degree of integration between the GO flakes and the ternary system.

These qualitative observations were further supported by helium pycnometry measurements (Table 4), which provided quantitative information on the density and porosity of the sponges. The incorporation of GO resulted in a marked increase in the density, from 0.21 ± 0.02 to 0.42 ± 0.01 g cm^−3^, with a corresponding decrease in total porosity from 82.5 ± 1.6% to 77.6 ± 1.2%. Although both formulations retained the highly porous architecture required for wound dressing applications, the scaffold containing GO exhibited a more compact internal structure. This behavior is consistent with the SEM observations, which revealed smaller pores and thicker pore walls in the sponges with GO. The increase in density and the concomitant reduction in porosity indicate that GO incorporation is associated with a more compact scaffold architecture. These structural changes may be part of the overall effect of GO incorporation and may contribute to the improved mechanical performance. However, the present study does not allow the contribution of these morphological changes to be quantitatively separated from potential GO-polymer interfacial effects.

**Table 4:** Density and porosity values of ChitCMCAlg and ChitCMCAlgGO

Sample	Density (g cm^−3^)	Porosity (%)
ChitCMCAlg	0.21 ± 0.02	82.5 ± 1.6
ChitCMCAlgGO	0.42 ± 0.01	77.6 ± 1.2

#### 3.1.4. Spectroscopic characterization of the composite dressings by FT-IR and solid-state NMR

The FT-IR spectra of Chit, CMC, Alg, and GO were recorded as reference materials prior to composite fabrication – Fig. 2A. Chit was dissolved in 2% (v/v) acetic acid, whereas CMC and Alg were prepared in distilled water.

**Fig. 2:**
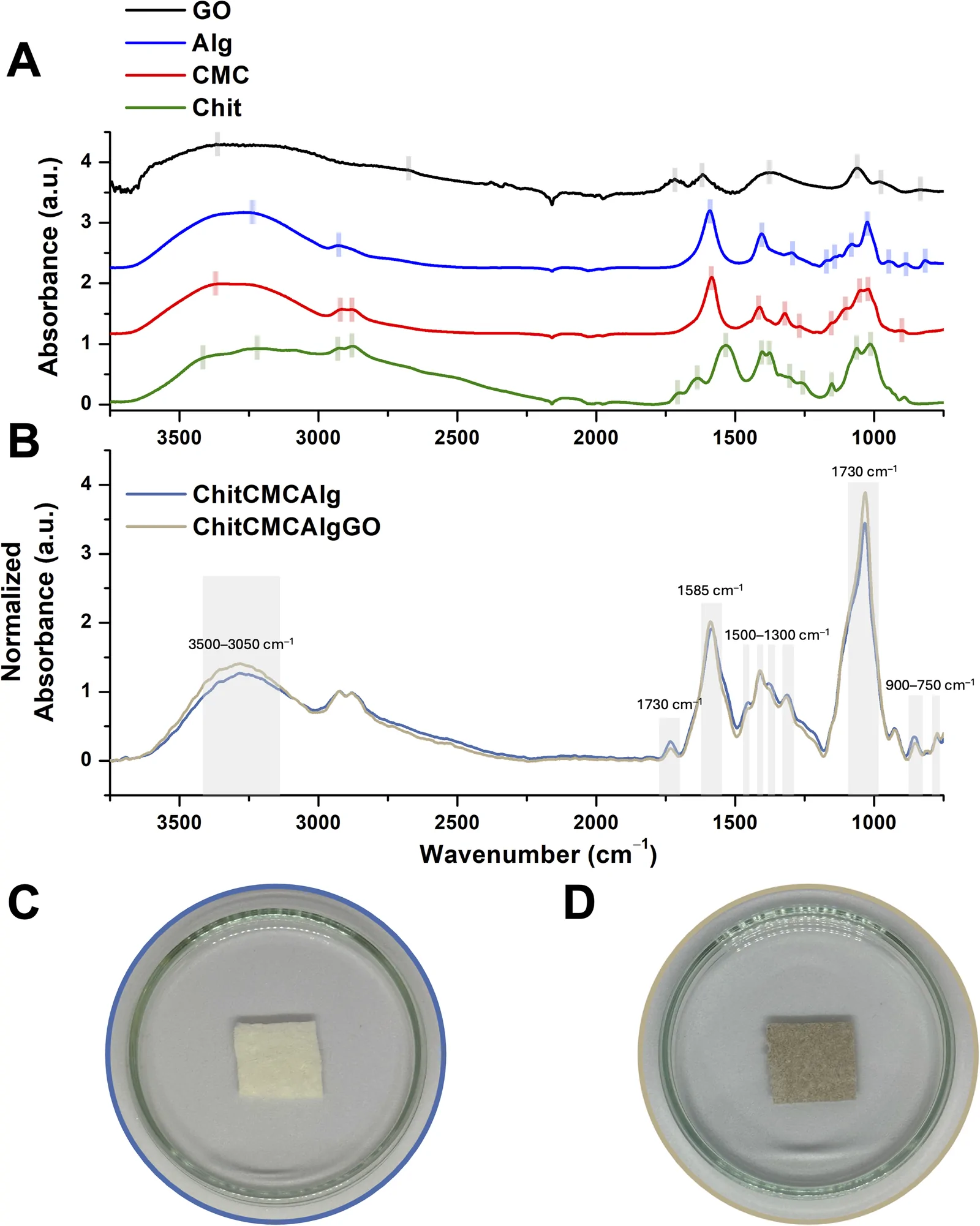
(A) FT-IR spectra of Chit, CMC, Alg, and GO recorded as reference materials, with characteristic bands marked: Chit (3395, 3204, 2929, 2873, 1710, 1640, 1537, 1408, 1375, 1305, 1253, 1152, 1064, 1015 cm^−1^), CMC (3275, 2919, 2874, 1585, 1415, 1321, 1265, 1154, 1103, 1050, 1021, 900 cm^−1^), Alg (3231, 2934, 1591, 1404, 1297, 1173, 1144, 1080, 1023, 938, 886, 816 cm^−1^), and GO (3346, 2690, 1717, 1616, 1383, 1061, 985, 832 cm^−1^). (B) FT-IR spectra of ChitCMCAlg matrices with and without GO, normalized to the band at ∼2930 cm^−1^. (C) Representative photograph of the composite material without GO. (D) Representative photograph of the material containing GO.

Chit exhibited a broad band at 3395–3204 cm^−1^, corresponding to overlapping O–H and N–H stretching vibrations. Bands at 2929 and 2873 cm^−1^ were assigned to asymmetric and symmetric C–H stretching vibrations. The signal at 1640 cm^−1^ is assigned to amide I vibrations (C=O stretching), while the band at 1537 cm^−1^ corresponds to N–H bending and C–N stretching (amide II). The weak band at 1710 cm^−1^ may be associated with residual acetyl groups and/or protonated carbonyl species formed under acidic conditions rather than covalent modification.^44^ Bands at 1408 and 1375 cm^−1^ are attributed to CH_2_ and CH_3_ bending vibrations. The region between 1305 and 1015 cm^−1^ (1305, 1253, 1152, 1064, 1015 cm^−1^) corresponds to C–O–C and C–O stretching vibrations of the polysaccharide backbone.^19,45^

CMC showed a broad O–H band at 3275 cm^−1^ and C–H stretching bands at 2919 and 2874 cm^−1^. The band at 1585 cm^−1^ was assigned to asymmetric stretching of carboxylate groups, and the band at 1415 cm^−1^ to symmetric COO^−^ stretching vibrations. Additional bands at 1321 and 1265 cm^−1^ correspond to O–H bending and C–O stretching vibrations, respectively. The strong signals between 1154–1021 cm^−1^ (1154, 1103, 1050, 1021 cm^−1^) are associated with C–O–C and C–O vibrations of the cellulose backbone, while the band at 900 cm^−1^ is related to β-glycosidic linkages.^46,47^

Alg exhibited a broad O–H stretching band at 3231 cm^−1^ and a C–H stretching band at 2934 cm^−1^. The dominant bands at 1591 and 1404 cm^−1^ correspond to asymmetric and symmetric stretching vibrations of carboxylate groups. The band at 1297 cm^−1^ is attributed to C–O stretching vibrations. The region between 1173–1023 cm^−1^ (1173, 1144, 1080, 1023 cm^−1^) corresponds to C–O–C and C–O vibrations of the polysaccharide backbone. Additional signals at 938, 886, and 816 cm^−1^ are associated with ring vibrations and mannuronic/guluronic acid residues, which are characteristic structural markers of Alg.^48^

GO showed a broad O–H band at 3346 cm^−1^ corresponding to O–H stretching vibrations of hydroxyl groups and adsorbed water molecules. The band at 1717 cm^−1^ is assigned to C=O stretching vibrations of carboxylic acid groups located at the edges of GO sheets. The band at 1616 cm^−1^ corresponds to skeletal vibrations of unoxidized graphitic domains (C=C stretching). The band at 1383 cm^−1^ is attributed to O–H bending vibrations. The absorption at 1061 cm^−1^ is associated with C–O stretching vibrations of epoxy and alkoxy groups, while the bands at 985 and 832 cm^−1^ correspond to C–O and C–H deformation modes within the oxidized graphene structure.^19^

The FT-IR spectra of ChitCMCAlg matrices with and without GO were analyzed to assess structural variations within the polymer network – Fig. 2B. The spectra were normalized with respect to the band at ∼2930 cm^−1^, corresponding to C–H stretching vibrations.

A noticeable increase in both intensity and broadening of the band in the 3500–3050 cm^−1^ region was observed for the GO-containing matrix. In the carbonyl region, the band at ∼1730 cm^−1^ was more pronounced in the matrix without GO and decreased upon GO incorporation. The band initially located at 1585 cm^−1^ exhibited a slight shift toward higher wavenumbers, accompanied by an increase in intensity in the presence of GO. Distinct differences were also observed in the 1500–1300 cm^−1^ region. The band at 1458 cm^−1^ was more intense in the matrix without GO, whereas the band at 1409 cm^−1^ showed increased intensity in the GO-containing sample. In addition, the band at 1373 cm^−1^ decreased in intensity after GO incorporation, while the band at ∼1318 cm^−1^ shifted toward higher wavenumbers and became less intense. A marked increase in intensity was observed at ∼1032 cm^−1^ for the GO-containing matrix. In the fingerprint region, the band at ∼857 cm^−1^ was more intense in the GO-free sample, whereas the band at ∼775 cm^−1^ became more pronounced in the presence of GO.

To further investigate the molecular organization of the polymer network after GO incorporation, solid-state NMR spectroscopy was performed. The solid-state ^13^C{^1^H} CP/MAS NMR spectra of ChitCMCAlg and ChitCMCAlgGO are presented in Fig. 3. Both spectra exhibit very similar resonance patterns, indicating that the incorporation of GO does not alter the primary chemical structure of the polymer matrix. No additional resonances or significant chemical shift changes were observed following GO incorporation, suggesting the absence of covalent interactions between GO and the constituent polymers.

**Fig. 3:**
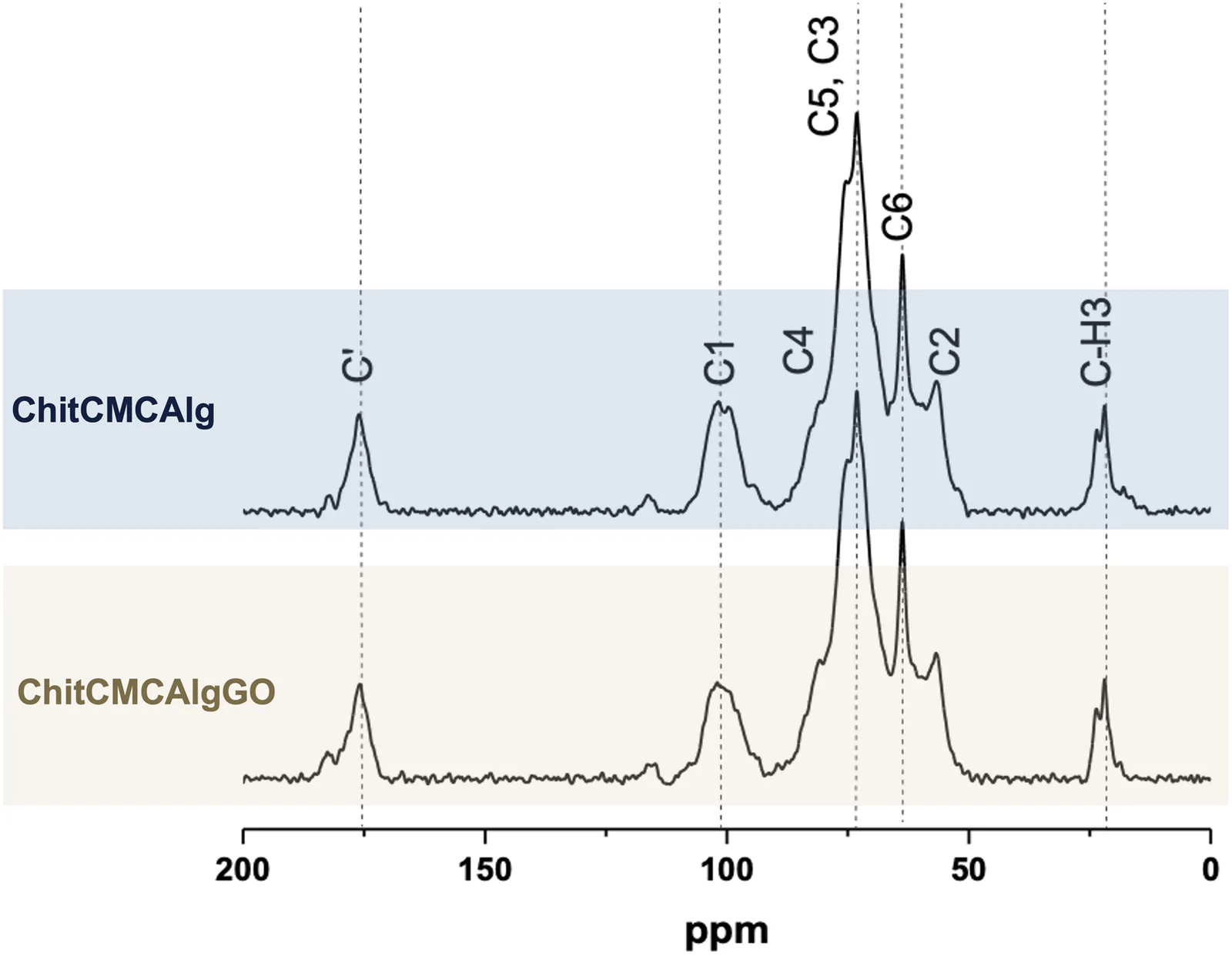
Solid-state ^13^C{^1^H} CP/MAS NMR spectra of the ChitCMCAlg and ChitCMCAlgGO sponge dressings. The spectra show the characteristic resonances of the polysaccharide backbone, including the anomeric carbon (C1), ring carbons (C2–C6), and the acetamide carbonyl (C′) and methyl (CH_3_) groups. The absence of significant chemical shift changes after GO incorporation indicates that the primary chemical structure of the polymer matrix remained unchanged.

The observed resonances were assigned to the carbon atoms of the β-(1 → 4)-linked polysaccharide backbone. The anomeric carbon (C1) appeared at approximately 101 ppm, while signals assigned to C2, C3, C4, C5, and C6 were detected in the expected spectral regions between 55 and 81 ppm. The resonance at approximately 175 ppm was attributed to the carbonyl carbon (C′) of the *N*-acetyl groups, whereas the signal at 22 ppm corresponded to the methyl carbon (CH_3_) of the acetamide moiety. The observed chemical shifts are in good agreement with previous reports for chitosan-based polysaccharides.^49^

Although the chemical shifts remained unchanged after GO incorporation, differences were observed in the proton and carbon spin–lattice relaxation times in the rotating frame (T_1_ρH and T_1_ρC), indicating modifications in the local molecular dynamics of the polymer network rather than changes in its chemical composition (Tables 5 and 6).

**Table 5:** T_1ρ_H relaxation time of ChitCMCAlg matrices with and without GO reported in function of chemical shift^a^

Chemical shift (ppm)	Peak	T_1ρ_H (µs) ChitCMCAlg	T_1ρ_H (µs) ChitCMCAlgGO
176	C′	0.6(3)	1.4(3)
102	C1	0.7(3)	1.2(3)
74	C5	0.7(3)	1.2(3)
64	C3	0.9(3)	1.2(3)
57	C2	0.9(3)	1.4(3)
22	CH_3_	—	—

a The error on the last digit is given in parentheses.

**Table 6:** T_1ρ_C relaxation time of ChitCMCAlg matrices with and without GO reported in function of chemical shift^a^

Chemical shift (ppm)	Peak	T_1ρ_C (µs) ChitCMCAlg	T_1ρ_C (µs) ChitCMCAlgGO
176	C′	16.3(6)	23.3(6)
102	C1	14.1(5)	12.2(5)
74	C5	8.2(3)	8.9(3)
64	C3	2.3(5)	4.3(3)
57	C2	4.5(6)	5.6(5)
22	CH_3_	2.7(4)	3.8(3)

a The error on the last digit is given in parentheses.

The T_1_ρH values of the GO-containing matrix were higher than those of the GO-free material for most of the analyzed carbon environments, suggesting differences in the local molecular dynamics of the polymer matrix following GO incorporation. However, T_1_ρ relaxation is influenced by molecular motions within a specific frequency regime, as well as by spin–diffusion effects and sample heterogeneity. Therefore, the observed changes cannot be interpreted as direct evidence of restricted polymer-chain mobility or used independently to establish specific polymer-GO interactions. The differences may reflect changes in the local molecular environment and segmental dynamics associated with GO incorporation. When considered alongside the FT-IR results, these findings are consistent with possible non-covalent interactions within the composite network, including hydrogen bonding and electrostatic contributions. However, these mechanisms cannot be conclusively established from the present data.

The T_1_ρC values also showed site-dependent changes following GO incorporation. The carbonyl carbon signal (C′, ∼176 ppm) exhibited an increase in T_1_ρC, whereas the C1 signal decreased, demonstrating that the relaxation response was not uniform across all carbon environments. These differences suggest changes in the local molecular environment and dynamics of selected functional groups but do not establish direct participation in specific intermolecular interactions. Further molecular-level studies would be required to distinguish the contributions of polymer-GO interactions from changes in matrix organization and heterogeneity.

Overall, the solid-state NMR results indicate that GO incorporation is associated with changes in the local molecular dynamics of the ChitCMCAlg matrix, without detectable changes in the principal chemical shifts of the polymer components. Together with the FT-IR and morphological findings, these results support the interpretation that GO incorporation affects the organization of the composite network. However, the relative contributions of specific non-covalent interactions and morphological effects to the observed mechanical reinforcement remain unresolved.

### 3.2. Antimicrobial and antibiofilm activity

#### 3.2.1. Antimicrobial activity against planktonic forms

The antimicrobial efficacy of the developed sponge dressings was evaluated against a broad spectrum of pathogens, including Gram-negative bacteria (*E. coli*, *P. aeruginosa*, *A. baumannii*), Gram-positive bacteria (*E. faecalis*, *S. aureus*), and the yeast-like fungus *C. albicans*. The results of the planktonic assays, expressed as the reduction in CFU mL^−1^, are summarized in Fig. 4.

**Fig. 4:**
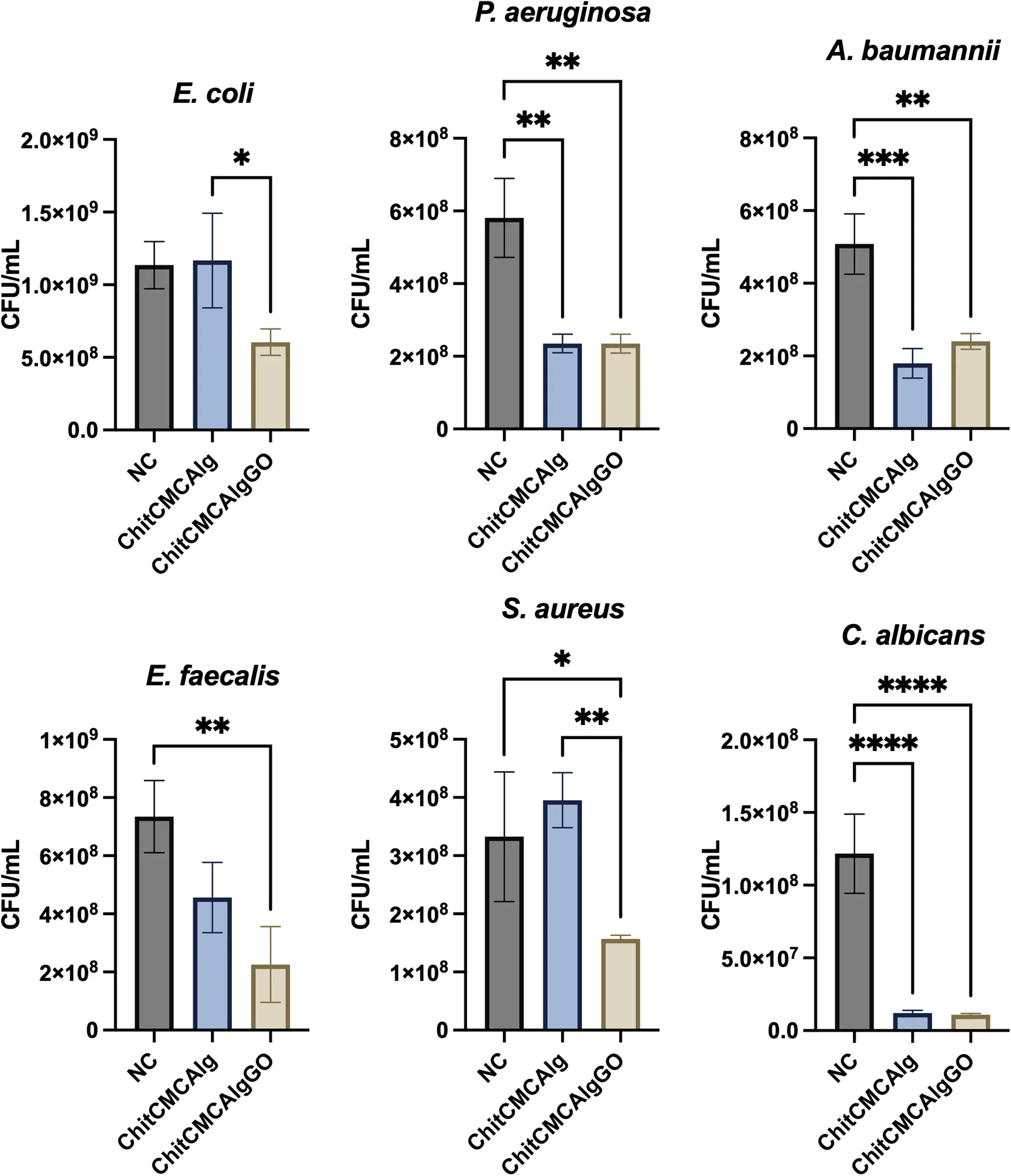
Comparative analysis of the antimicrobial activity of the developed sponge dressings against planktonic forms of selected pathogens. The results are expressed as the CFU mL^−1^ for the following groups: negative control (NC), unmodified matrix (ChitCMCAlg), and GO-functionalized matrix (ChitCMCAlgGO). Data are presented as mean values ± standard deviation (SD). Statistical significance was determined using one-way ANOVA followed by Tukey's HSD *post hoc* test. Asterisks indicate significant differences between groups: *p ≤ 0.05, **p ≤ 0 0.01, ***p ≤ 0.001, and ****p ≤ 0.0001.

Statistical analysis revealed that the antimicrobial response was highly strain dependent. The strongest inhibitory effect was observed against *C. albicans*, where both the ChitCMCAlg and ChitCMCAlgGO showed a highly significant reduction in microbial population compared to the negative control (*p* < 0.0001). High effectiveness was also demonstrated against Gram-negative bacteria: *A. baumannii* (*p* = 0.0007) and *P. aeruginosa* (*p* = 0.0010). For these strains, *post hoc* Tukey tests confirmed significant differences for both types of dressings relative to the control (*p* < 0.01).

In the case of Gram-positive bacteria, the materials significantly inhibited the growth of *S. aureus* with the GO-containing matrix showing slightly higher statistical significance (*p* = 0.0048) than the unmodified one (*p* = 0.0223). Interestingly, for *E. faecalis*, a statistically significant reduction compared to the control was achieved only after the incorporation of GO (*p* = 0.0060), suggesting that the carbon filler enhances the matrix's performance against this specific pathogen.

Regarding *E. coli*, the ANOVA indicated a statistically significant difference across the groups (*p* = 0.0325). While the reduction relative to the NC did not reach the significance threshold in the *post hoc* analysis, a significant difference was observed between the ChitCMCAlg and ChitCMCAlgGO dressings (*p* = 0.0434). This finding indicates that GO incorporation may improve the antibacterial performance of the matrix against *E. coli* under the tested conditions. However, this result alone does not establish a broader antimicrobial spectrum.

Overall, both the ChitCMCAlg and ChitCMCAlgGO matrices exhibited antimicrobial activity, with the effect of GO incorporation varying among the tested microorganisms. Although GO incorporation was associated with improved activity against selected strains, the present results do not establish a general broadening of the antimicrobial spectrum.

The capacity of the developed sponge dressings to eradicate pre-established, 24-hour mature biofilms was evaluated using a static model. The results, expressed as the survival of microbial cells (CFU mL^−1^) after contact with the dressings, are presented in Fig. 5. Like the results obtained in the planktonic assays, the antibiofilm activity was strain-dependent, though the complex structure of the biofilm matrix increased the resistance of several pathogens.

**Fig. 5:**
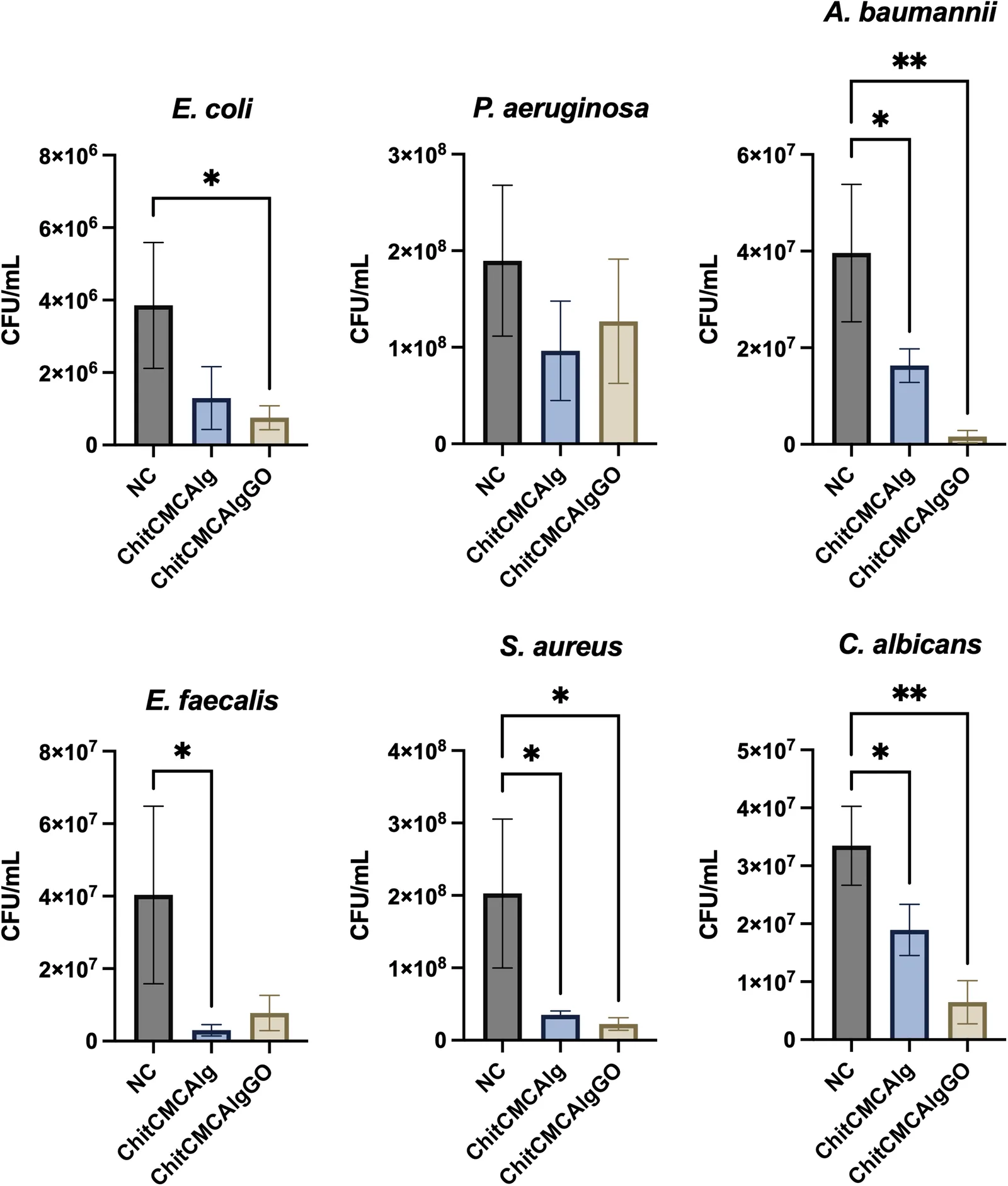
Antibiofilm activity of the sponge dressings against mature biofilms established on polystyrene surfaces. Results are expressed as the CFU mL^−1^ for the following groups: negative control (NC), unmodified matrix (ChitCMCAlg), and GO-functionalized matrix (ChitCMCAlgGO). Data represent mean values ± SD. Statistical significance was determined using one-way ANOVA followed by Tukey's HSD *post hoc* test. Asterisks indicate significant differences between groups: *p ≤ 0.05, **p ≤ 0.01.

The most pronounced reduction in viable, culturable cells counts was observed for *C. albicans* (one-way ANOVA, *p* = 0.0021), where both the unmodified ChitCMCAlg dressing (*p* = 0.0315) and the functionalized ChitCMCAlgGO dressing (*p* = 0.0017) significantly reducing the number of recoverable viable cells compared with the negative control. A significant reduction was also observed for *A. baumannii* (one-way ANOVA, *p* = 0.0044), with both materials demonstrating antimicrobial activity (*p* = 0.0348 and *p* = 0.0037, respectively).

In the case of Gram-positive bacteria, the dressings were effective against *S. aureus* (one-way ANOVA, *p* = 0.0177), with both types of sponges achieving significant biofilm eradication. For *E. faecalis*, however, only the unmodified ChitCMCAlg matrix showed a statistically significant reduction compared to the control (*p* = 0.0445).

Regarding Gram-negative rods, the incorporation of GO proved crucial for activity against *E. coli*, while the overall one-way ANOVA was significant (*p* = 0.0331), only the ChitCMCAlgGO dressing achieved a statistically significant reduction (*p* = 0.0361) in the *post hoc* analysis. Notably, *P. aeruginosa* exhibited the highest resistance in the static biofilm model (one-way ANOVA, *p* = 0.2812), with no significant reduction observed for any of the tested materials, highlighting the protective nature of the extracellular polymeric substances produced by this strain.

Despite the increased resistance associated with mature biofilm formation, both sponge formulations retained substantial antibiofilm activity against several clinically relevant pathogens. Particularly strong effects were observed against *A. baumannii*, *S. aureus*, and *C. albicans*, highlighting the potential applicability of the developed materials in the management of biofilm-associated chronic wound infections.

#### 3.2.2. Spatial biofilm eradication and permeability assessment

The A.D.A.M. model was employed to evaluate the penetration depth of active agents through multiple layers of mature biofilm. The results, expressed as the percentage reduction in microbial metabolic viability, revealed distinct differences in the performance of the unmodified and GO-functionalized dressings (Fig. 6).

**Fig. 6:**
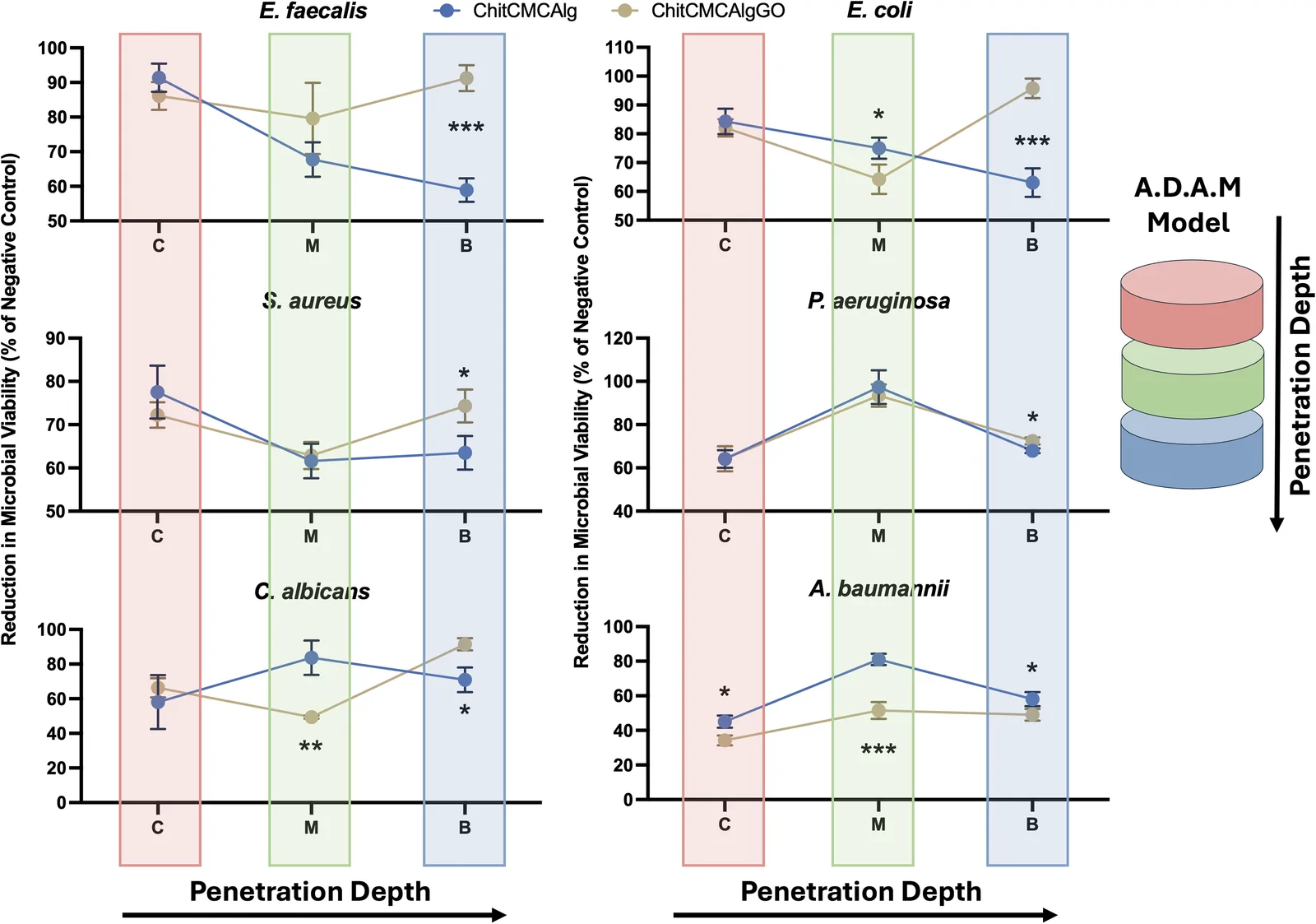
Spatial antibiofilm efficacy of the dressings evaluated *via* the A.D.A.M. model. The graph illustrates the reduction in microbial viability (% of negative control) across three vertical layers of biofilm: C (contact/top), M (medium/middle), and B (basal/bottom). Data represent mean values ± SD. Statistical significance between ChitCMCAlg and ChitCMCAlgGO was determined using multiple unpaired *t*-tests (with *p* ≤ 0.05 considered significant). Asterisks indicate significant differences between the two types of dressings: *p ≤ 0.05, **p ≤ 0.01, ***p ≤ 0.001. The schematic diagram on the right illustrates the multi-layered agar disc setup and the direction of antimicrobial agent penetration from the dressing through the biofilm structure.

Across most tested strains, the top layer (C), which was in direct contact with the materials, showed high and comparable reduction rates for both dressings, except for *A. baumannii*, where the GO-modified matrix significantly outperformed the raw blend (*p* = 0.0144). Interestingly, in the middle layer (M), the incorporation of GO provided a significant advantage against *C. albicans* (*p* = 0.0041), *E. coli* (*p* = 0.0412), and *A. baumannii* (*p* = 0.0009), suggesting that GO may facilitate the sustained release or diffusion of antimicrobial components through the biofilm matrix.

However, in the basal layer (B) – the furthest from the dressing – a shift in efficacy was observed. For *S. aureus*, *E. faecalis*, *E. coli*, and *P. aeruginosa*, the unmodified ChitCMCAlg matrix demonstrated significantly higher metabolic reduction compared to the GO-containing composite. This suggests that while GO reinforces the matrix and enhances activity in the upper layers, the denser structural network and the strong intermolecular interactions evidenced in FTIR may slightly slow down the diffusion of active agents to the deepest layers in this specific spatial model. The exception remained *A. baumannii*, where the ChitCMCAlgGO dressing maintained superior activity throughout all three layers, including the basal disc (*p* = 0.0408).

Altogether, the A.D.A.M. model revealed distinct depth-dependent antimicrobial behaviors of the tested materials, indicating that GO functionalization influences not only antimicrobial potency but also the spatial diffusion profile within mature biofilm structures.

### 3.3. Biocompatibility studies

#### 3.3.1. Confirmation of the high biocompatibility of composite materials in the indirect cytotoxicity assay

The cytocompatibility of the ChitCMCAlg matrices with and without GO was evaluated using an extract-based assay in accordance with ISO 10993-5.

For both materials, cell viability exceeded the 70% threshold defined by the ISO 10993-5 standard, indicating a lack of cytotoxicity – Fig. 7A. In the case of the GO-free matrix, statistical analysis confirmed normal distribution and homogeneity of variance. One-way ANOVA revealed significant differences among groups (*p* = 0.0005). *Post hoc* comparisons showed a statistically significant increase in cell viability at 100% extract concentration compared to the control (*p* = 0.0016), while no significant differences were observed for lower extract concentrations. Similarly, extracts obtained from the GO-containing matrix did not exhibit cytotoxic effects. One-way ANOVA with Dunnett's *post hoc* test indicated a statistically significant overall effect (*p* = 0.0368), however, pairwise comparisons with the control group did not reveal significant differences for individual extract concentrations (*p* > 0.05), including the highest concentration (*p* = 0.0659).

**Fig. 7:**
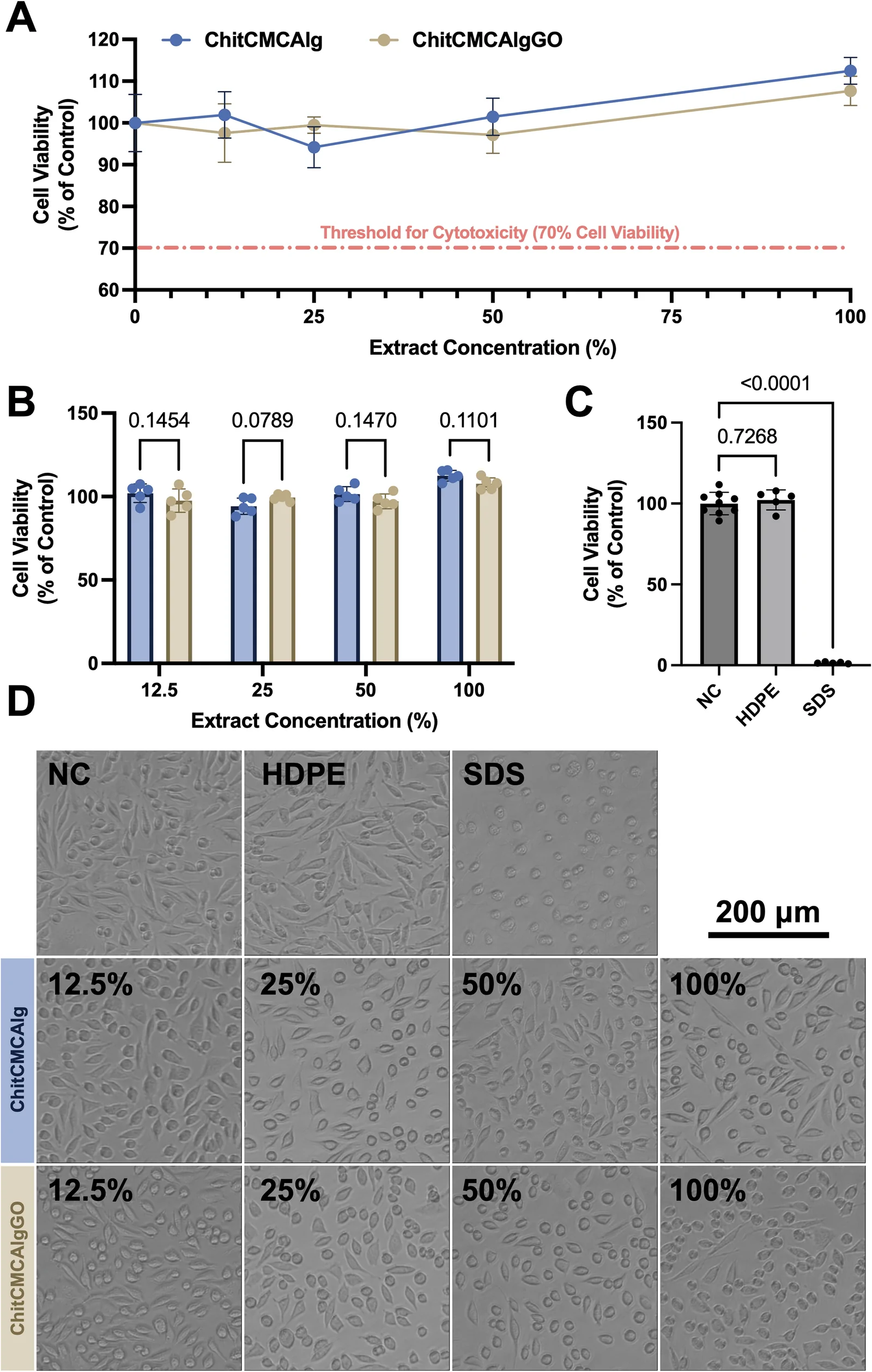
(A) Dose–response relationship of L929 cell viability after exposure to different concentrations of material extracts (12.5%, 25%, 50%, and 100%) of ChitCMCAlg matrices with and without GO, expressed as percentage of the negative control. (B) Statistical comparison of cell viability between matrices with and without GO at corresponding extract concentrations. (C) System validation (system response) showing comparison of negative control (NC), high-density polyethylene (HDPE) extract, and positive cytotoxic control (SDS). (D) Representative phase-contrast micrographs of L929 cells after exposure to material extracts (12.5%, 25%, 50%, and 100%), as well as controls (NC, HDPE, and SDS). Scale bar: 200 µm.

Direct comparison between matrices with and without GO using Student's *t*-test showed no statistically significant differences at any extract concentration (*p* > 0.05), indicating comparable cytocompatibility profiles – Fig. 7B.

The assay system was validated using appropriate controls. No significant difference was observed between the negative control and HDPE extract (*p* = 0.7268), confirming non-cytotoxic conditions. In contrast, a highly significant reduction in cell viability was observed for the SDS-treated group (*p* < 0.0001), confirming proper assay sensitivity – Fig. 7C.

Morphological assessment of L929 cells exposed to material extracts revealed normal cell morphology, with well-spread fibroblast-like cells and preserved membrane integrity – Fig. 7D. According to ISO 10993-5 grading criteria, the observed cellular response corresponded predominantly to grade 0 (no reactivity) and occasionally grade 1 (slight reactivity). Cells exhibited normal morphology, with no evidence of cell lysis, significant vacuolization, or detachment. Only minor and sporadic changes were observed, such as discrete intracellular granules or slight alterations in cell shape, without any notable inhibition of cell growth.

#### 3.3.2. Confirmation of the high biocompatibility of composite materials in the direct cytotoxicity assay

The cytocompatibility of the ChitCMCAlg matrices with and without GO was also evaluated using a direct cytotoxicity assay in accordance with ISO 10993-5.

Following 24 h of direct contact with the tested materials, L929 cells maintained normal morphology both in proximity to and at a distance from the material surface. No signs of cytotoxic effects were observed, including changes in cell shape, detachment, membrane disruption, or reduced cell density. The cell monolayer remained continuous and comparable to the control group, indicating that the materials do not exert local cytotoxic effects under direct contact conditions – Fig. 8A.

**Fig. 8:**
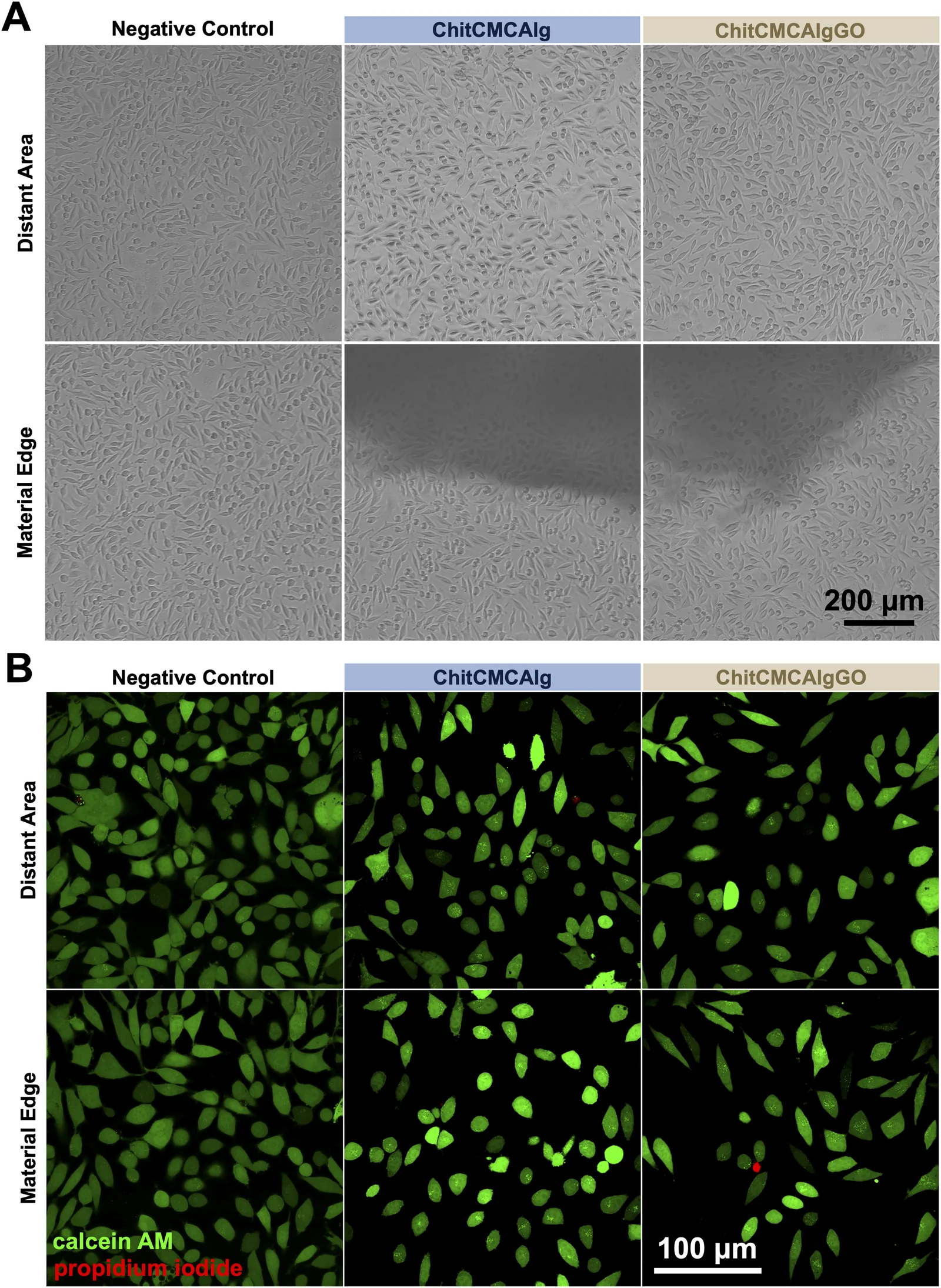
(A) Phase-contrast microscopy images of L929 cells under direct contact conditions, showing cell morphology in the distant area and at the material edge. Scale bar: 200 µm. (B) Live/dead fluorescence staining (calcein AM/propidium iodide) of L929 cells in the distant area and at the material edge after direct contact exposure, showing viable (green, calcein-positive) and non-viable (red, PI-positive) cells. Scale bar: 100 µm.

Fluorescence imaging using calcein AM and PI staining confirmed high cell viability in both tested systems. Strong green fluorescence (calcein-positive viable cells) was observed both in the vicinity of the material and in more distant regions of the cell layer. No significant red fluorescence (PI-positive dead cells) was detected, indicating intact cell membranes and absence of cytotoxic damage – Fig. 8B.

## 4 Discussion

Chronic wounds represent a complex clinical challenge in which impaired tissue regeneration, excessive inflammation, persistent oxidative stress, and microbial biofilm formation interact to prevent effective healing.^7^ In this context, current wound management strategies increasingly emphasize antimicrobial stewardship and the reduction of unnecessary antibiotic exposure. According to international recommendations, including those of the European Wound Management Association, systemic antibiotics should be restricted primarily to clinically infected wounds, whereas many chronic wounds require optimized local wound management strategies based on advanced biomaterials capable of controlling the wound microenvironment.^50,51^ Therefore, the development of multifunctional dressings that simultaneously provide structural support, regulate exudate, maintain a moist environment, and exhibit antimicrobial and antibiofilm activity represents an important direction in modern biomaterials research.^52^

In the present study, a chitosan–carboxymethylcellulose–alginate (ChitCMCAlg) sponge was functionalized with graphene oxide (GO) to generate a multifunctional composite dressing with enhanced mechanical stability and antimicrobial performance while preserving biological compatibility. The obtained results demonstrate that GO acts not merely as an antimicrobial additive but as a structural modulator capable of reorganizing the polymeric network at multiple hierarchical levels, including molecular interactions, pore architecture, mechanical behavior, and biological performance.

The substantial improvement in mechanical properties following GO incorporation indicates the formation of an efficiently reinforced polymeric network. GO nanosheets possess a high specific surface area and abundant oxygen-containing functional groups, including hydroxyl, epoxy, and carboxyl groups, enabling extensive interactions with polysaccharide chains.^28,53^ These interactions, primarily involving hydrogen bonding and electrostatic forces, enhance interfacial adhesion between GO and the surrounding polymer matrix, facilitating more effective stress transfer during mechanical deformation.^54,55^ Similar reinforcement mechanisms have been reported for GO-containing polysaccharide-based biomaterials, where GO functions as a nanostructural scaffold rather than a conventional passive filler.^56–59^

Importantly, the simultaneous increase in tensile strength and elongation at maximum stress demonstrates that GO incorporation does not result in excessive matrix rigidification or brittle behavior, which is frequently observed in nanocomposite systems.^60,61^ Instead, the obtained data suggest the development of a mechanically reinforced yet flexible network. This behavior is particularly relevant for wound dressing applications, where materials must withstand manipulation during application and removal while maintaining intimate contact with irregular wound surfaces.^62–64^ Thus, GO-mediated reinforcement provides an advantageous balance between durability and conformability, two properties that are often difficult to achieve simultaneously in highly porous biomaterials.

The mechanical improvement was accompanied by a moderate reduction in sorption capacity, reflecting a structural trade-off associated with GO-induced network densification. The incorporation of GO promotes additional intermolecular interactions that restrict excessive polymer chain mobility and reduce matrix expansion during hydration. Consequently, the accessibility of hydrophilic functional groups and free volume available for water penetration become partially limited. However, this reduction in swelling capacity should not be interpreted as a functional disadvantage. Excessive fluid uptake may compromise the mechanical integrity of highly hydrated scaffolds, leading to uncontrolled swelling, loss of shape stability, and difficulties during clinical handling.^65–67^ The mechanical properties reported in this study were determined under the specified testing conditions and may not fully represent the behavior of the dressings in a hydrated wound environment. Since water uptake can alter the structural integrity and mechanical response of polymer-based materials, further testing under wet or simulated wound conditions is required to assess their mechanical stability during practical use.

From a biomedical perspective, the ideal wound dressing requires not maximal absorption alone, but rather controlled fluid management combined with preservation of structural integrity. The GO-containing composite developed in this study demonstrates such optimization, maintaining therapeutically relevant absorption capacity while exhibiting substantially improved mechanical robustness. This balance may be particularly beneficial in chronic wounds characterized by prolonged exudation, where mechanical degradation of the dressing may limit treatment effectiveness.

Although water sorption is an important parameter in the characterization of wound-dressing materials, the results obtained in this study should be interpreted as an initial assessment of the hydration behavior of the developed composites under the tested conditions. Water uptake alone does not establish their ability to manage wound exudate under clinical conditions, as practical performance also depends on fluid retention, mechanical integrity in the hydrated state, moisture-vapor transmission, and interactions with the wound environment. Therefore, the observed sorption properties indicate the potential of these materials for further development but do not, by themselves, establish their clinical suitability. Further evaluation under simulated wound conditions is warranted.

The SEM observations provide direct morphological evidence supporting the functional changes induced by GO incorporation. The transition from a highly open porous structure toward a more refined and interconnected pore architecture suggests that GO promotes controlled organization of the polymer matrix rather than simple pore reduction. This interpretation is further supported by helium pycnometry analysis, which demonstrated increased density and reduced total porosity while maintaining a highly porous structure. Therefore, GO induces structural refinement rather than pore collapse, preserving the interconnected network required for fluid transport, oxygen diffusion, and potential cellular infiltration.^68,69^ The relationship between porosity and mechanical properties in the developed composites should be interpreted in the context of their overall structure. Although increased porosity can reduce the effective load-bearing cross-section and influence mechanical strength, the resulting mechanical response also depends on pore size and distribution, matrix continuity, and interactions between the constituent polymers and graphene oxide. Consequently, differences in porosity and mechanical performance should not be interpreted as evidence of a direct causal relationship. Further studies combining quantitative porosity analysis with mechanical testing under controlled conditions would be required to establish the relative contribution of these structural factors.

The associations between morphological, mechanical, and physicochemical properties suggest that the effects of GO incorporation occur at multiple structural levels. At the molecular level, interactions between GO and the polysaccharide components may contribute to interfacial reinforcement. At the microscale, the accompanying changes in pore size, pore-wall morphology, density, and porosity may also influence the mechanical response of the scaffold. These effects are not mutually exclusive and may jointly contribute to the observed improvement in tensile performance. However, the present study does not allow their individual contributions to be quantitatively distinguished.

FT-IR analysis provided spectroscopic evidence of changes in the local chemical environment following GO incorporation, which may be consistent with non-covalent interactions between GO and the polysaccharide components. The observed changes in hydroxyl, amino, and carboxyl-associated spectral regions indicate the formation of an extended intermolecular interaction network between GO and polysaccharide components. The broadening and increased intensity of the O–H/N–H stretching region suggest enhanced hydrogen bonding, resulting from interactions between oxygenated GO functionalities and hydroxyl, amino, and carboxyl groups present in Chit, CMC, and Alg. Similar spectral modifications have been previously associated with improved interfacial compatibility in GO-reinforced polysaccharide composites.^70–72^

The redistribution of signals within the carbonyl and carboxylate regions indicates changes in the local chemical environment and possible modification of COOH/COO^−^ equilibrium.^57–59^ Importantly, these changes do not indicate chemical modification of the polymer backbone but rather reflect physical interactions and charge redistribution within the composite network. This conclusion is supported by ss-NMR analysis, where the absence of substantial chemical shift changes confirms preservation of the molecular identity of the constituent polymers.^49,73^ Overall, the FT-IR and solid-state NMR findings are consistent with changes in the local molecular environment following GO incorporation, without evidence of major changes in the chemical structure of the constituent polymers. Although non-covalent interactions and physical confinement may contribute to the observed reinforcement, their relative importance cannot be established from the present data alone.

Such preservation of the native polymer structure is particularly important for biomedical applications, as it allows retention of the inherent biological properties of chitosan, cellulose derivatives, and alginate while introducing additional functional advantages provided by GO. The resulting composite therefore combines the biological functionality of naturally derived polymers with the structural benefits of nanomaterial reinforcement.

Beyond structural improvement, the developed materials demonstrated clinically relevant antimicrobial and antibiofilm activity against wound-associated pathogens. Importantly, the antimicrobial response was strain-dependent, reflecting differences in microbial envelope structure, metabolic activity, and biofilm organization. The highest susceptibility was observed for *C. albicans* and *A. baumannii*, whereas *P. aeruginosa* demonstrated greater resistance, particularly in mature biofilm models. This behavior is consistent with the highly organized extracellular polymeric matrix and intrinsic adaptive mechanisms characteristic of mature *P. aeruginosa* biofilms.^74^

GO incorporation was associated with improved antimicrobial performance against selected microorganisms, although the magnitude of the effect varied depending on the strain and experimental model. The observed activity may involve multiple mechanisms, including interactions between GO nanosheets and microbial membranes, membrane disruption, oxidative stress, and effects on biofilm architecture. However, these mechanisms were not directly investigated in the present study. Importantly, the observed activity should not be attributed exclusively to GO, as the polysaccharide matrix itself contributes antimicrobial properties, particularly through electrostatic interactions associated with chitosan-based systems.^75,76^

The A.D.A.M. biofilm model additionally demonstrated that antimicrobial effectiveness depends not only on microbial susceptibility but also on spatial distribution of active components within the biofilm structure.^41^ The different penetration profiles observed for GO-containing and GO-free materials suggest that GO remains strongly associated with the polymer network, limiting extensive migration while maintaining activity at the wound interface. Such immobilization may represent an important safety advantage by reducing the risk of uncontrolled nanomaterial release into surrounding tissues.^77,78^

The cytocompatibility results further support the biomedical potential of the developed composites. Both ChitCMCAlg and GO-containing dressings fulfilled ISO 10993-5 requirements, demonstrating high fibroblast viability after indirect and direct exposure. Importantly, GO incorporation did not compromise biological safety, indicating that the nanosheets were effectively integrated within the polymeric matrix and did not generate significant cytotoxic effects.

This finding is particularly relevant because the biological response of GO-based biomaterials depends strongly on its concentration, dispersion state, and immobilization within the host matrix. Free or aggregated GO structures may induce cellular stress responses, whereas GO incorporated into a stable polymer network can provide functional enhancement while maintaining cytocompatibility.^75,76,79^ The preserved fibroblast morphology and favorable live/dead staining results indicate that the developed composites provide an appropriate biological environment compatible with tissue repair processes.

The cytocompatibility assessment was conducted in accordance with the relevant ISO 10993 framework. Nevertheless, cytocompatibility is only one component of the biological evaluation of medical devices, and the present results should not be interpreted as establishing comprehensive biological safety for clinical use.

Recent developments in wound-dressing research increasingly focus on multifunctional materials that combine antimicrobial protection with the ability to influence the wound microenvironment. Polysaccharide-based systems incorporating GO have been investigated for a range of biomedical applications, including antimicrobial materials and functional hydrogel platforms.^80^ Other advanced approaches, such as polysaccharide-based smart and electrically active dressings, illustrate the broader transition towards materials designed to support more complex wound-management strategies.^81^ In particular, the regulation of macrophage responses and the transition from prolonged inflammation towards tissue repair are recognized as important targets in the development of bioactive wound dressings.^82^ In the present study, the ChitCMCAlgGO composite was evaluated in terms of its physicochemical characteristics, antimicrobial performance, and cytocompatibility. These findings provide an initial characterization of the material. However, direct effects on immune-cell responses, cell proliferation, angiogenesis, and wound regeneration were not investigated and therefore cannot be inferred from the present results.

Overall, the present study demonstrates that GO incorporation enables the transformation of a conventional polysaccharide sponge into a multifunctional wound dressing platform with improved mechanical stability, controlled hydration behavior, enhanced antimicrobial activity, and preserved cytocompatibility. Rather than acting solely as an antimicrobial additive, GO incorporation was associated with changes in scaffold architecture, mechanical performance, hydration behavior, and antimicrobial activity. The observed changes in structural and functional properties may reflect the combined effects of GO incorporation, its interactions with the polysaccharide matrix, and the resulting modifications in scaffold morphology. These findings highlight the potential of GO-reinforced natural polymer systems as advanced biomaterials for the management of infected and hard-to-heal wounds, where mechanical reliability, microbial control, and biological safety must be achieved simultaneously.

## 5 Conclusions

GO likely acts primarily as a multifunctional structural nanofiller that reorganizes the molecular architecture of the chitosan–carboxymethylcellulose–alginate network through non-covalent interactions rather than altering the chemical structure of the constituent polysaccharides.

The GO-induced molecular reorganization resulted in a denser and mechanically reinforced scaffold with preserved porosity, demonstrating that modulation of polymer-chain dynamics represents an effective strategy for improving the structural performance of polysaccharide-based wound dressings.

Structural densification was accompanied by a moderate reduction in fluid uptake, indicating a functional trade-off between exudate absorption and mechanical stability while maintaining fluid-handling properties suitable for chronic wound management.

GO incorporation enhanced antimicrobial and antibiofilm performance, particularly against Gram-negative bacteria and *Candida albicans*, while preserving broad-spectrum antimicrobial activity and cytocompatibility in accordance with ISO 10993.

The established relationship between molecular organization, scaffold architecture, and biological performance provides mechanistic insight into the design of GO-reinforced polysaccharide biomaterials and supports their further development as advanced wound dressings for infected chronic wounds.

## Author contributions

Przemysław Sareło: conceptualization, data curation, formal analysis, funding acquisition, investigation, methodology, project administration, resources, validation, visualization, writing – original draft, writing – review & editing. Maria Wiśniewska-Wrona: conceptualization, data curation, formal analysis, investigation, methodology, writing – review & editing. Monika Sikora: conceptualization, data curation, formal analysis, investigation, methodology, writing – review & editing. Magdalena Pajączkowska: investigation, methodology, resources, writing – review & editing. Joanna Nowicka: investigation, methodology, resources, writing – review & editing. Yuriy Gerasymchuk: resources. Maria Luisa Saladino: data curation, investigation, methodology. Patrizia Cancemi: data curation, investigation, methodology. Martina Maria Calvino: data curation, investigation, methodology. Giuseppe Lazzara: data curation, investigation, methodology. Maria Szymonowicz: conceptualization, methodology, project administration, resources, supervision, writing – review & editing. Beata Sobieszczańska: conceptualization, funding acquisition, supervision, writing – review & editing. Magdalena Wawrzyńska: conceptualization, funding acquisition, project administration, supervision, writing – review & editing.

## Conflicts of interest

The authors declare that they have no known competing financial interests or personal relationships that could have appeared to influence the work reported in this paper.

## Abbreviations

Alg: alginate
Chit: chitosan
ChitCMCAlg: chitosan–carboxymethyl cellulose–alginate composite
ChitCMCAlgGO: chitosan–carboxymethyl cellulose–alginate composite with graphene oxide
CMC: carboxymethylcellulose
GO: graphene oxide

## Data Availability

The data that support the findings of this study are available from the corresponding author upon reasonable request.
